# Selective Noradrenaline Depletion in the Neocortex and Hippocampus Induces Working Memory Deficits and Regional Occurrence of Pathological Proteins

**DOI:** 10.3390/biology12091264

**Published:** 2023-09-21

**Authors:** Chiara Prinzi, Anna Kostenko, Gioacchino de Leo, Rosario Gulino, Giampiero Leanza, Antonella Caccamo

**Affiliations:** 1 Department of Drug and Health Sciences, University of Catania, 95125 Catania, Italy; chiara27chiara@gmail.com; 2 B.R.A.I.N. (Basic Research and Integrative Neuroscience) Laboratory for Neurogenesis and Repair, Department of Life Sciences, University of Trieste, 34100 Trieste, Italy; akostenka@gmail.com; 3 SISSA, Scuola Internazionale Superiore di Studi Avanzati, 34136 Triste, Italy; deleo.gioacchino@gmail.com; 4 Department of Biomedical and Biotechnological Sciences, University of Catania, 95125 Catania, Italy; rosario.gulino@unict.it; 5 Molecular Preclinical and Translational Imaging Research Centre-IMPRonTE, University of Catania, 95125 Catania, Italy; 6 Department of Chemical, Biological, Pharmaceutical and Environmental Sciences, University of Messina, 98168 Messina, Italy

**Keywords:** locus coeruleus, Alzheimer’s disease, TDP-43, Tau, cognitive impairment, rat

## Abstract

**Simple Summary:**

Noradrenaline is a crucial neurotransmitter produced by a group of neurons in the locus coeruleus and plays a significant role in regulating various physiological processes, including attention, arousal and stress responses. Alzheimer’s disease is a progressive and devastating neurodegenerative disorder that affects millions of people worldwide. It is characterized by the accumulation in the brain of abnormal protein aggregates, such as amyloid-beta, tau and TDP-43, leading to the deterioration of neurons and cognitive decline. Studies have shown that the noradrenergic system is severely affected by Alzheimer’s disease. This depletion of noradrenaline levels has been associated with the cognitive decline and behavioral symptoms observed in Alzheimer’s patients. In this study, we showed that administering increasing doses of a selective noradrenergic immunotoxin caused dose-dependent working memory impairments and accumulation of TDP-43 phosphorylated at Ser 409/410 and Tau phosphorylated at Thr 217 in the cortex and hippocampus of developing rats. These findings suggest that boosting noradrenergic activity may have beneficial effects on cognitive functions and slow down disease progression. However, further research is needed to fully comprehend the complex relationship between noradrenaline, cognitive function and the aberrant accumulation of proteins in Alzheimer’s disease and to develop effective treatments based on these findings.

**Abstract:**

Noradrenaline (NA) depletion occurs in Alzheimer’s disease (AD); however, its relationship with the pathological expression of Tau and transactive response DNA-binding protein 43 (TDP-43), two major hallmarks of AD, remains elusive. Here, increasing doses of a selective noradrenergic immunotoxin were injected into developing rats to generate a model of mild or severe NA loss. At about 12 weeks post-lesion, dose-dependent working memory deficits were detected in these animals, associated with a marked increase in cortical and hippocampal levels of TDP-43 phosphorylated at Ser 409/410 and Tau phosphorylated at Thr 217. Notably, the total levels of both proteins were largely unaffected, suggesting a direct relationship between neocortical/hippocampal NA depletion and the phosphorylation of pathological Tau and TDP-43 proteins. As pTD43 is present in 23% of AD cases and pTau Thr217 has been detected in patients with mild cognitive impairment that eventually would develop into AD, improvement of noradrenergic function in AD might represent a viable therapeutic approach with disease-modifying potential.

## 1. Introduction

Alzheimer’s disease (AD) is the most common form of dementia worldwide. Clinically, it is characterized by a progressive loss of cognitive skills, such as memory, executive functions and language. In addition, local accumulations of several misfolded proteins such as amyloid-β (Aβ), hyperphosphorylated Tau and transactive response DNA-binding protein 43 (TDP-43) in various brain areas represent major histopathological hallmarks of the disease [1]. The Aβ peptide is a cleavage product of the amyloid precursor protein (APP) and is the main component of senile plaques in the brain of AD patients [2,3]. The Tau protein belongs to the microtubule-associated protein (MAP) family, and it is produced by alternative splicing from the MAPT gene [4]. Physiologically, Tau stabilizes microtubules. However, it undergoes hyperphosphorylation, and this post-translational modification reduces its affinity for microtubules, thereby impairing axonal transport [5]. Hyperphosphorylated Tau aggregates and forms neurofibrillary tangles (NTFs) [6]. Several amino acids of the Tau sequence are known to be phosphorylated, and the phosphorylation status of many of these sites has been associated with the progression of the disease. Recently, Tau phosphorylated at Thr 217 (T217) has raised particular interest as several groups have reported that is a highly specific marker for detecting both preclinical and advanced AD. The occurrence of Tau T217 in the cerebrospinal fluid (CSF) is predictive of AD pathology in asymptomatic and symptomatic stages [7,8,9].

TDP-43 is encoded by the TAR DNA Binding Protein (TARDBP) gene [10]. It regulates gene expression, the splicing and turnover of RNAs and the biogenesis of miRNAs. Hyperphosphorylated, i.e., pathological, TDP-43 forms cytoplasmic inclusions detectable in frontotemporal dementia (FTD) and amyotrophic lateral sclerosis (ALS), as well as in 36–56% of AD cases [1,11,12]. There is also evidence that TDP-43 directly interacts with Aβ and Tau; in fact, some studies have reported colocalization between TDP-43, plaques and tangles [13,14]. Phosphorylated TDP-43 is more prone to aggregation [15]; in fact, TDP-43 phosphorylated at Ser 409/410 and 403/404 accumulates in AD brains [16].

The locus coeruleus (LC) is the main source of noradrenergic innervation in the central nervous system and projects to the neocortex and hippocampus, i.e., brain regions closely involved in cognition and memory formation and storage [17]. Alterations in tonic and phasic neuronal activity in the noradrenergic system may lead to memory dysfunction [18,19]. Loss of LC neurons is believed to start during the transition from no cognitive impairment (NCI) to mild cognitive impairment (MCI) and progresses in the late AD stages [20,21]. In normal conditions, noradrenaline (NA) also regulates phagocytosis, a neuroprotective process that clears pathological proteins [22]. However, its deficiency impairs this mechanism, leading to the accumulation of pathological proteins [23,24,25]. Thus, neuronal loss in the LC might contribute to the detection of increased levels of Tau T217 and T181 in the CSF and blood of patients with AD [7,9,26]. Tau manifests at the early stages of AD in LC, and from there it spreads throughout the brain as the disease progresses [27,28]. Converging evidence indicates that early alterations in the structure and function of the noradrenergic system contribute to the neuropathological alterations observed in rodent models of AD [29,30]. Modeling these changes in experimental animals would certainly be of great value in the quest for viable therapeutic strategies. However, neurotransmitter dysfunctions and the accumulation of pathological proteins have often been investigated independently. There is scant preclinical evidence about the possible relationships between altered amyloid expression in the neocortex/hippocampus and reduced neurotransmission, the latter generally referring to cholinergic inputs from the basal forebrain neurons [31,32,33,34,35,36]. Interestingly, recent evidence indicates that early alterations in the structure and function of the noradrenergic system may also affect amyloid expression in rodent models of AD [29,30]. However, not much experimental work has addressed the possible effects of early noradrenergic loss on other neuropathological hallmarks of AD, such as regional expression of hyperphosphorylated Tau and/or TDP-43. We have previously shown that depletion of LC noradrenergic neurons, induced by a selective immunotoxin, severely affects specific cognitive abilities and markedly increases the cytoplasmic occurrence of TDP-43 in hippocampal neurons, a possible index of its pathological transformation [37]. In the present study, we sought to follow up on those findings and examined the effects of selective noradrenergic depletions on spatial learning and memory functions and tissue expression of phosphorylated (i.e., pathological) TDP-43 and Tau proteins in cognitively relevant regions such as the neocortex and hippocampus.

## 2. Materials and Methods

### 2.1. Animals and Experimental Design

Twenty-four evenly distributed male and female Sprague–Dawley rats, obtained from three different litters at the animal facility of the University of Trieste, were utilized for this study. Pups were kept with their mothers until reaching 21 days of age, housed in double-decker cage units (Tecniplast, Buggiate, VA, Italy) and maintained under standard conditions of light, temperature and humidity, with unrestricted access to food and water. On the 4th day after birth (P4), the rat pups were assigned randomly to groups injected with a low dose (LD, n = 8) or a high dose (HD, n = 8) of a selective noradrenergic immunotoxin, the anti-dopamine β -hydroxylase (DBH)-saporin. An additional group of pups underwent similar injections but received phosphate-buffered saline (PBS) instead (sham-lesioned, n = 4). The remaining pups did not undergo any surgical procedures and were used as unoperated controls (intact, n = 4).

The assessment of sensory-motor and cognitive abilities commenced at 6 and 12 weeks post-lesion, respectively. Following the completion of these evaluations, the rats were euthanized, and their brains were processed for post-mortem analyses. All the experiments adhered to the Italian Guidelines for Animal Care (D.L. 116/92 and 26/2014) and were conducted in agreement with the directives outlined in 2010/63/EU.

### 2.2. Lesion

The lesion procedures were carried out as previously described [38]. Briefly, pups under hypothermic anesthesia were injected bilaterally with 0.2 µg (0.1 μg/side; LD) or 0.4 µg (0.2 μg/side; HD) of anti-DBH-saporin (Advanced Targeting Systems, San Diego, CA, USA), dissolved in 5 + 5 μL sterile PBS, into the lateral ventricles at the following coordinates (in mm, relative to bregma and outer skull surface): AP = −0.6, L = ±0.8, V = −2.1, using a 10 μL Hamilton microsyringe (Hamilton, Bonaduz, Switzerland) at a speed of 2 µL/min, allowing 3 min for diffusion before retracting the cannula to reduce backflow. Sterile PBS was injected into sham-lesioned animals using the same volume, coordinates and speed. After each surgery, pups were allowed to fully recover and reacquire normal body temperature under a filament bulb, prior to being returned to the mothers and left undisturbed.

### 2.3. Behavioral Tests

All the behavioral tests were performed between 9:00 a.m. and 3:00 p.m. To assess potential non-specific motor disturbances that might have been triggered by the immunotoxin, the animals were subjected to simple motor tests (bridge test and grid test) to specifically evaluate limb strength and coordination, as previously reported [39,40]. In the bridge test, rats were placed onto a narrow wooden beam (70 cm long and 2 cm wide), connected to their home cage. The time spent with all four limbs on the beam and the latency to cross the beam were recorded. In the grid test, rats were placed facing downwards onto a 50° inclined wire mesh screen (100 × 100 cm), connected to their home cage. The latency to rotate upwards and move toward their cage and the number of falls from the grid were recorded.

Morris water maze. The Morris water maze (MWM) is a widely adopted behavioral task developed to evaluate spatial learning and memory in rodents [41]. The main testing apparatus is a circular pool 150 cm in diameter and 50 cm deep, filled with room-temperature water. The rats were released into the pool from four equally spaced points, indicated as North, South, East and West, and the pool was divided into 4 quadrants (SW, NW, NE, SE), each containing a central circular area (annulus) where an escape platform would be anchored if placed in that quadrant. The pool was in a room containing many visual cues that may be used by the animals to find a platform placed in the center of one quadrant 2 cm below the water surface. Starting from 12 weeks post-lesion, the animals were first allowed to swim freely for 60 s, to become accustomed to the pool, followed by a three-day cued training session. Throughout this period, the platform’s visibility was facilitated by a striped flag, and its location was changed for each of the four trials conducted each day. The point of the test was to exclude non-cognitive (e.g., visual) impairments that may affect the execution of the spatial version of the test. After the cued test, animals were tested in the reference memory version of the MWM.

Rats received 4 trials a day, with a 30 s inter-trial interval, for 7 consecutive days, and in each trial, they were released from a different starting point and allowed to swim for 60 s to reach the hidden platform, always kept in the SW quadrant. If the platform was not found, the animal was manually led by the experimenter to the platform and left on it for 30 s. After the fourth trial of the last day of testing, a probe trial was administered. The platform was removed, and the latency and distance required by the animals to locate the platform in a single 60 s trial, as well as the swim distribution in the various quadrants during the spatial probe trial, were recorded using a computer-based video tracking system.

Radial arm water maze. The radial arm water maze (RAWM) is a modified procedure of MWM, developed to evaluate working memory [42,43]. It consists of the same pool as above, where 6 swim alleys (50 cm length × 20 cm wide), radiating out of an open central area, were created. An escape platform was located at the end of one of the alleys and remained in the same location for the 5 trials on the same day. The platform was moved to a new arm every day over the 5 consecutive days of testing. At the beginning of each trial, the animal was released from one of the unbaited arms (i.e., an arm without the platform) and had 60 s to find the hidden platform, with a 30 s inter-trial interval.

If the animal was not able to find the platform, it was led to the platform and left on it for 30 s. The latency to reach the platform and the arm selection errors (i.e., visiting an arm that did not contain the platform or an already visited arm) in each trial were recorded and averaged across days. The data were also analyzed in terms of “savings”, defined as performance improvements between the first and second trials, according to the following formula:

Savings = (t1 − t2)/t1 × 100

where t1 is the average latency (or the average number of errors) to find the hidden platform in the first trial and t2 is the average latency (or the average number of errors) in the second trial.

### 2.4. Post-Mortem Analyses

After the behavioral tests, 16-week-old rats were sacrificed, and their brains were removed. The frontoparietal cortex (cortex) and the hippocampus from one hemisphere were dissected and stored at −80 °C for Western blot analyses. The remaining part of the brain was first fixed for about 24h in ice-cold 4% paraformaldehyde in phosphate buffer (PB, pH 7.4) and then kept at 4 °C in a 20% sucrose solution until it was cut with a freezing microtome (Leitz Welzlar, Wetzlar, Germany), for immunohistochemistry and quantitative morphometric analyses.

Immunohistochemistry. Changes in noradrenergic neuronal and fiber distribution induced by the low or high dose of anti-DBH-saporin were visualized by Dopamine- -hydroxylase (DBH) immunohistochemistry, using a standard avidin-biotin ABC procedure on a series of sections cut from the hemisphere not taken for Western blot assay, encompassing the frontoparietal cortex and the hippocampus to the LC level [37,40,44].

Briefly, after endogenous peroxidase activity was quenched with 10% methanol and 3% H_2_O_2_ in 0.02 M potassium phosphate-buffered saline (KPBS, pH 7.4), the sections were pre-incubated for 1h in blocking buffer (5% normal horse serum (and 0.3% Triton in KPBS), and then they were exposed overnight to mouse monoclonal anti-DBH primary antibody (1:2000; Merck Millipore, Burlington, MA, USA) in 2% NHS and 0.3% Triton in KPBS. The sections were rinsed with KPBS and incubated for 2h at room temperature with horse anti-mouse biotinylated secondary antibody (1:200, Thermo Fisher Scientific, Waltham, MA, USA) in 2% NHS and 0.3% Triton in KPBS, followed by a one-hour incubation in avidin-biotin-peroxidase solution (ABC kit, Vector Laboratories, Burlingame, CA, USA). The staining was visualized using 0.025% 3,3′-diaminobenzidine (Sigma-Aldrich, St. Louis, MI, USA) as a chromogen and 0.01% hydrogen peroxide in KPBS for 2–5 min. The sections were mounted on chromalum-gelatin-coated slides, dehydrated through steps in ascending alcohol concentrations, clarified in xylene and coverslipped using DPX (Sigma-Aldrich, St. Louis, MI, USA).

Microscopic analysis and quantitative evaluation. The degree of reduction in noradrenergic neurons caused by the lesion was estimated using an unbiased stereological quantification technique based on the optical fractionator principle [45]. DBH-immunoreactive neurons in the LC were counted bilaterally from the level of the ventral portion of the parabrachial nucleus, rostrally, to the genu of the facial nerve, caudally. The counting also included the more scattered noradrenergic neurons in the subcoeruleus region, just ventral to the LC proper [46,47].

For this process, an Olympus BH2 microscope with an X-Y motorized stage and a microcator for Z-axis measurements was used. This was linked to a color video camera (Sony) and a personal computer. The CAST GRID^®^ 2.0 software from Olympus Denmark A/S was used to outline the designated regions at 4× magnification. Additionally, the software generated unbiased counting frames, which were systematically and randomly moved until the entire demarcated region had been sampled. Positive cells were identified and counted using a 100× oil objective, and guard volumes were excluded from both sides of the section surfaces to avert issues of lost caps.

Lesion-induced changes in the density of cortical and hippocampal noradrenergic innervation were assessed using the Image 1.61 NIH image analysis software [48]. Measurement fields of consistent size (0.5 mm in diameter) were chosen bilaterally at comparable rostrocaudal levels from three different sections in the frontoparietal cortex, the CA1 and CA3 subregions of the dorsal hippocampus, and the dentate gyrus. The background density, determined in a region lacking the DBH-positive reaction product (such as the corpus callosum), was subtracted from each measurement. The resulting data were presented in arbitrary units, representing the average values obtained from the analysis of the three sections.

Western blots. The Western blot analyses to evaluate the steady-state levels of TDP-43 and Tau were performed according to a previously reported protocol [49]. Briefly, cortices and hippocampi were homogenized in ice-cold RIPA buffer (Thermo Fisher Scientific, Waltham, MA, USA) with the addition of protease and phosphatase inhibitors and then centrifuged at 12,000 rpm for 30 min at 4 °C. Supernatants were then collected and stored at −80 °C. Protein extracts were loaded in a 4–12% polyacrylamide precast gel (Thermo Fisher Scientific, Waltham, MA, USA). After separation, proteins were transferred to a nitrocellulose membrane, using the Trans-Blot SD Semi-Dry transfer cell (Biorad, Hercules, CA, USA). The membrane was incubated for one hour in 5% non-fat dry milk in TBS-T (0.1% Tween 20, 100 mM Tris pH 7.5, 150 mM NaCl) and then kept exposed overnight at 4 °C to the specific primary antibody. The day after, membranes were rinsed three times with TBS-T, incubated for one hour with a fluorescent secondary antibody and then rinsed three times with TBS-T. The protein bands were visualized and quantified using LI-COR Odyssey (LI-COR, Lincoln, NE, USA). The following primary antibodies were used: Rabbit anti-TDP-43 (1:1000; Proteintech, Rosemont, IL, USA), Mouse anti-TDP-43 phosphorylated at Ser 409/410 (1:200; Proteintech, Rosemont, IL, USA), Rabbit anti-GAPDH (1:7000; Cell Signalling, Danvers, MA, USA), Mouse anti-Tau (1:2000; Immunological Sciences, Roma, Italy), Rabbit anti-Tau phosphorylated at Thr 217 (1:200; Immunological Sciences, Roma, Italy). Secondary antibodies were as follows: goat anti-mouse IRDye 680LT or goat anti-rabbit IRDye 800CW (1:10,000; LI-COR, Lincoln, NE, USA). Some cortical and hippocampal samples from the various groups were lost due to technical problems. Western blot analyses were therefore conducted on the remaining tissue specimens (Supplementary Materials).

### 2.5. Statistical Analysis

Data fulfilled the criteria for normal distribution and were therefore analyzed using parametric tests for all statistical comparisons. Group differences in behavioral performance as well as in cell numbers, innervation density or protein levels were assessed by either one-way analysis of variance (ANOVA) or two-way mixed ANOVA as appropriate, followed by the Tukey HSD post hoc test. Data are presented as means ± standard error of the mean (Sem), and differences were considered significant at *p* < 0.05.

## 3. Results

### 3.1. Central Noradrenaline Depletion Has No Effects on Sensory-Motor Activity

**Figure 1:**
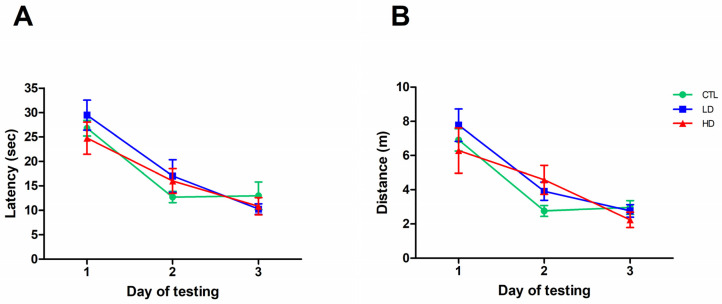
The immunotoxin lesion does not cause sensory impairments. Escape latency (**A**) and swim distance (**B**) recorded in the cued version of the MWM. Each point represents the mean value ± standard error of the mean (SEM) for the 4 trials administered on each of the 3 days of testing.

All rats, irrespective of the lesion, had a steady increase in body weight and exhibited normal sensory-motor functioning in the bridge and grid tests (Table 1). Intact and sham-lesioned rats did not differ in any behavioral, morphological or biochemical parameters; therefore, they were combined into a single control group (CTL, n = 8) for all analyses and illustrations. Animals’ performance in the cued version of the Morris water maze (MWM) test, administered at about 12 weeks post-lesion, with the platform made visible to rule out possible lesion-induced sensory-motor impairments, is shown in Figure 1A,B. Overall, the rats in the various groups improved their performance over time (two-way mixed ANOVA, effect of day on latency, F2,42 = 46.24; on distance, F2,42 = 46.89; both *p* < 0.001) and did not differ from each other (main group effect on latency, F2,21 = 0.27; on distance, F2,21 = 0.28; group × day on latency, F4,42 = 1.18; on distance, F4,42 = 1.23; all *p* > 0.05, n.s.). Thus, no unspecific deficit was induced by the lesion that would affect navigation ability in the pool.

### 3.2. Central Noradrenergic Depletion Does Not Affect Reference Memory

**Figure 2:**
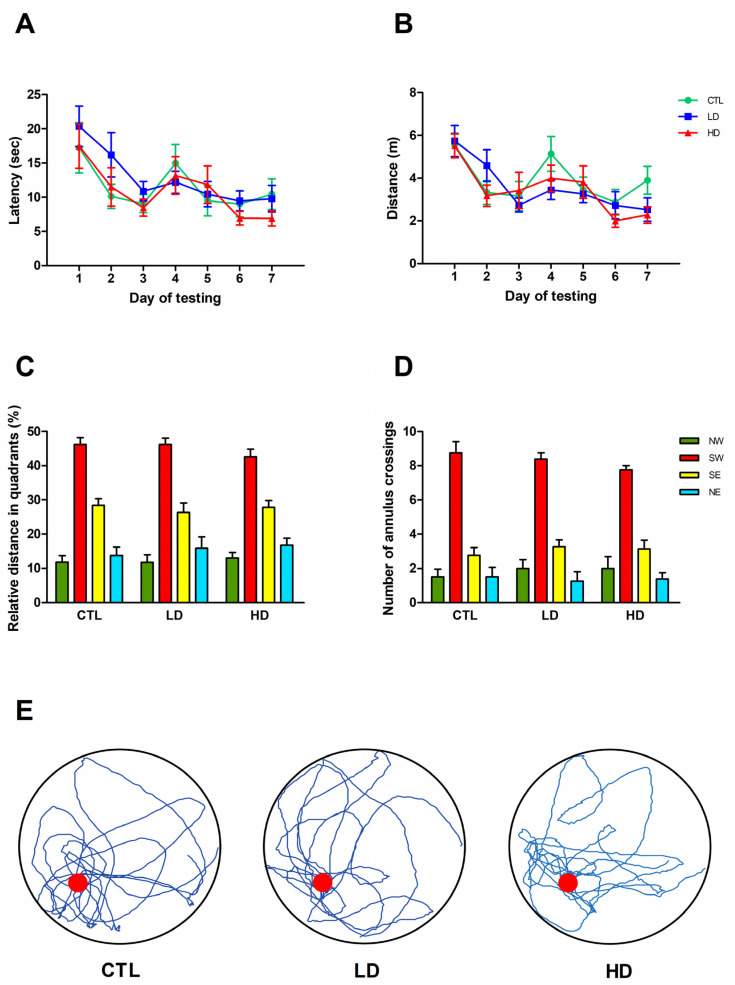
Noradrenergic depletion does not affect reference memory performance. Escape latency (**A**) and swim distance (**B**) recorded in the MWM task. Each point represents the mean value ± standard error of the mean (SEM) for the 4 trials on each of the 7 days of testing. The relative distance percentage swum in quadrants (**C**), the number of annulus crossings (**D**) and the swim paths of representative rats of CTL, LD and HD groups (**E**), recorded during the spatial probe trial are illustrated. The red dot in (**E**) represents the platform position in the pool.

Groups’ performances in the reference memory version of the MWM are shown in Figure 2A–E. In the place test, conducted over seven days with four trials a day, all animals learned to locate the hidden platform and significantly reduced the required time and distance over time (two-way mixed ANOVA, effect of day on latency, F6,126 = 9.98; on distance, F6,126 = 10.52; both *p* < 0.001, Figure 2A,B). Again, the groups did not differ from each other, (main group effect on latency, F2,21 = 0.42; on distance, F2,21 = 0.52; group × day on latency, F12,126 = 0.8; on distance F12,126 = 1.19; all *p* > 0.05, Figure 2A,B). At the end of the fourth trial of the last day of testing, the platform was removed, and animals were administered a fifth spatial probe trial to assess the strength of the previous learning. In general, all animals exhibited a marked bias for the original site where the platform was located and concentrated their swim mainly in the SW (training) quadrant. Two-way mixed ANOVA revealed a significant effect of quadrant both for the distance and annulus crossing measures (F3,63 = 97.79 and F3,63 = 97.79, respectively; both *p* < 0.001), but the groups did not show any difference (main group effect on distance, F2,21 = 0.91; on annulus crossings, F2,21 = 0.15; group × quadrant interaction on distance, F6,63 = 0.41; on annulus crossings, F6,63 = 0.48; all *p* > 0.05, Figure 2C,D). When evaluated together with the actual swim paths from representative animals (Figure 2E), these findings indicated that noradrenergic depletion has no significant effect on reference memory abilities.

### 3.3. Lesion-Induced Noradrenergic Depletion Causes Working Memory Deficits in a Dose-Dependent Manner

**Figure 3:**
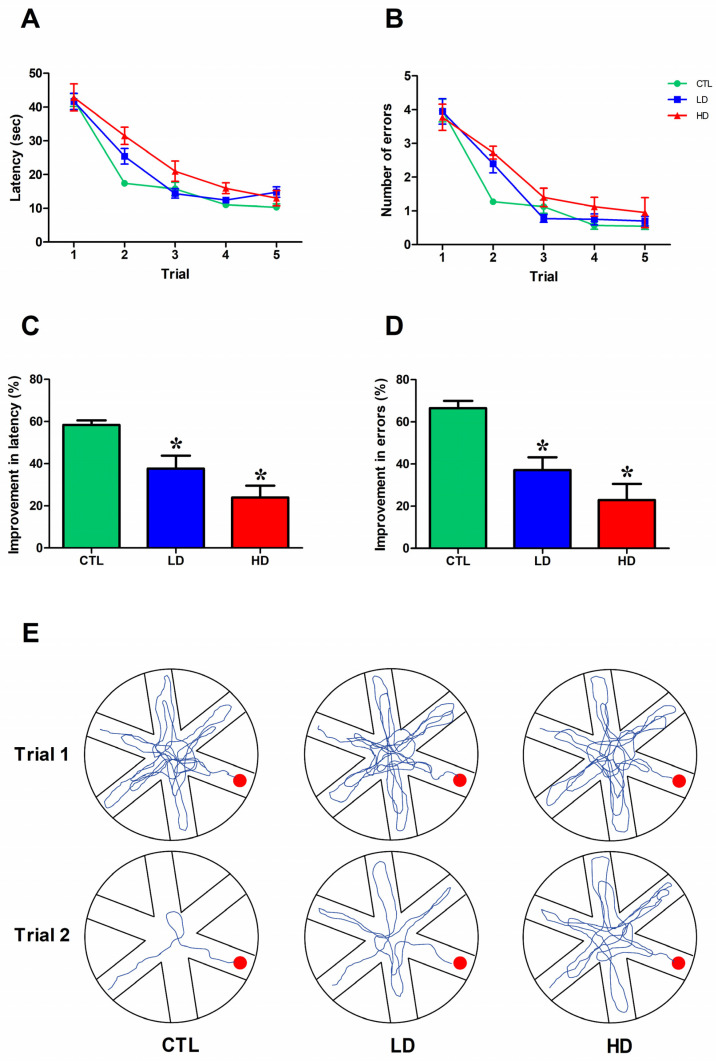
Noradrenergic depletion impairs working memory performance. Escape latency (**A**) and number of errors (**B**) recorded in the RAWM task. Each point represents the mean value ± standard error of the mean (SEM) for the 5 trials on each of the 5 days of testing. Performances are also plotted as percentage of savings between trials 1 and 2; percentage of improvement in latency (**C**) and errors (**D**) and the representative swim paths of rats from CTL, LD and HD groups (**E**) are illustrated. The red dot in (**E**) represents the platform position in the pool. Asterisks indicate significant differences from CTL group (*p* < 0.05).

Figure 3A–E illustrates animals’ performances in the RAWM task, administered at about 14 weeks post-lesioning to evaluate the effects of the treatments on working memory function. Overall, the animals had longer latencies and made more arm selection errors during the first trial on each day. They then progressively reduced time and errors over the five trials (two-way mixed ANOVA, effect of trials on latency, F4,84 = 124.16; on errors, F4,84 = 88.29; both *p* < 0.001); however, such improvements varied among groups (group × trial interaction on latency, F8,84 = 2.43; on errors, F8,84 = 2.08; all *p* < 0.05), likely due to the poor performance of the two lesioned groups, which were less efficient compared to CTL (main group effect on latency, F2,21 = 5.43; on errors, F2,21 = 3.47; one-way ANOVA + Fisher’s PLSD post hoc test for both measures, *p* < 0.05) (Figure 3A,B). Latency and error savings, calculated as the percent improvements between trials 1 and 2, provided a further screen of animals’ performance in the RAWM. Thus, whereas the CTL group exhibited a ≈60–65% improvement in both measures, the LD and HD groups were significantly less efficient; their improvements never exceeded 35–40% and 20–25%, respectively (one-way ANOVA + Tukey post hoc test; *p* < 0.05 for both measures vs. CTL; Figure 3C,D). Inspection of the actual swim paths from representative animals in the groups (Figure 3E) confirmed the dose-dependent nature of the working memory deficits, associated with the increasing severity of the noradrenergic depletion.

### 3.4. Increasing Doses of Anti-DBH-Saporin Injected in the Lateral Ventricles of P4 Rats Produce a Dose-Dependent Loss of Noradrenergic Neurons and Fibers

**Figure 4:**
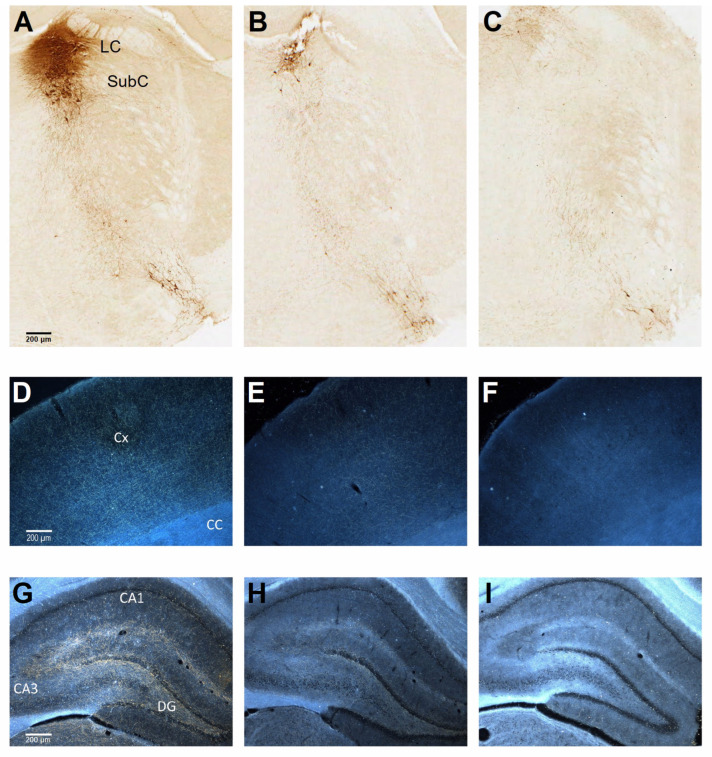
The anti-DBH-saporin injection induces noradrenergic neuron and fiber loss in a dose-dependent manner. (**A**–**C**) Representative examples of DBH immunostaining illustrating, in bright field, the appearance of the LC/SubC complex (**A**–**C**), and in dark field, the appearance of the frontoparietal cortex (**D**–**F**) and hippocampus (**G**–**I**) in coronal sections, from CTL, LD and HD animals. Note in (**C**–**I**) the almost complete loss of immunoreactive neurons and fibers of the HD group and the somewhat milder depletion exhibited by the LD animals compared to CTL. Cx, cortex; CC, corpus callosum; CA, cornu ammonis; DG, dentate gyrus. Scale bar in **A** (for **A**–**C**), **D** (for **D**–**F**) and **G** (for **G**–**I**): 200 μm.

The bilateral intraventricular injection of a low dose (0.2 μg/side, LD) or high dose (0.4 μg/side, HD) of anti-DBH-saporin produced, at about 4 months post-lesion, a marked, dose-dependent depletion of DBH-immunoreactive neurons in the LC/SubC complex (Figure 4A–C). As estimated by stereology (Table 2), the loss of noradrenergic neurons averaged about 71% and 90% in the animals treated with a low and a high dose of the immunotoxin, respectively, compared to CTL, and these lesion groups also differed from one another (one-way ANOVA, followed by Tukey post hoc test; for all comparisons, *p* < 0.01).

Both groups of lesioned animals exhibited a dramatic loss of noradrenergic innervation throughout the frontoparietal cortex and in all hippocampal subfields. The fiber depletion appeared virtually complete in the specimens from HD animals (Figure 4F,I), whereas in those from the LD group, some spared fibers could be detected (Figure 4E,H), as opposed to CTL (Figure 4D,G), indicating a clear dose-related pattern of lesion-induced fiber depletion. Semi-quantitative estimations of the relative levels of DBH-positive innervation density in the neocortical and hippocampal regions are illustrated in Table 2. Statistical comparisons confirmed a dramatic 64–71% and 86–90% DBH-positive fiber depletion in the animals from the LD and HD groups, respectively, compared to CTL, and the lesion groups also differed from each other (two-way ANOVA, followed by Tukey post hoc test; for all comparisons, *p* < 0.01).

### 3.5. High Dose of Anti-DBH-Saporin Leads to an Increase in TDP-43 Phosphorylated at Ser 409/410 in the Cortex

**Figure 5:**
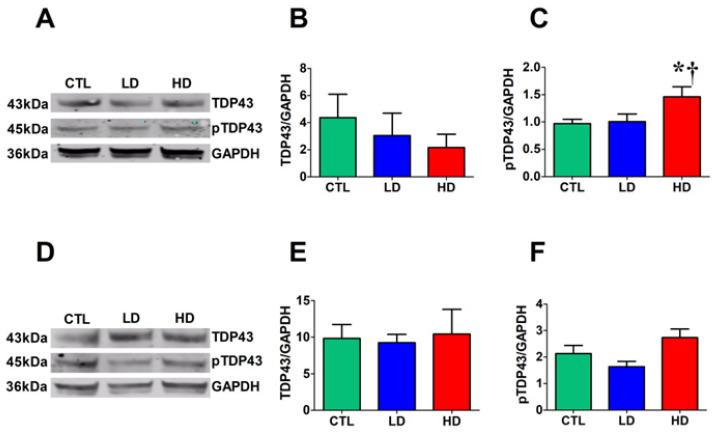
Increased levels of pTDP-43 in cortex of HD rats. (**A**) Representative Western blot bands of proteins extracted from the cortex of the 3 groups. Densitometric analysis of TDP-43 (**B**) and pTDP-43 (**C**) steady-state levels of proteins extracted from the cortex. (**D**) Representative Western blot bands of proteins extracted from the hippocampus of the 3 groups. Densitometric analysis of TDP-43 (**E**) and pTDP-43 (**F**) steady-state levels of proteins extracted from the hippocampus. Data were obtained by normalizing our protein of interest to GAPDH loading control. Asterisk and dagger indicate significant differences from CTL and LD groups, respectively (*p* < 0.05).

In a previous study, a highly significant correlation was found between the depletion of noradrenergic fibers in the hippocampal dentate gyrus (DG) and the cytoplasmic mislocalization of TDP-43 immunoreactivity in DG neurons [37]. Here, to determine whether the noradrenergic lesion caused an aberrant increase in the steady-state levels of TDP-43, the tissue levels of total TDP-43 were first measured, followed by the analysis of the levels of TDP-43 phosphorylated at Ser 409/410 in all groups. One-way ANOVA and the Tukey post hoc test revealed that the total TDP-43 levels in either the cortex (Figure 5A,B) or the hippocampus (Figure 5D,E) did not differ among groups. By contrast, the levels of phosphorylated TDP-43 in the cortex and hippocampus of the HD group were increased (by about 50%) compared to the CTL and LD groups, but only the former was significant (*p* < 0.05; Figure 5A–C), whereas the hippocampal increase failed to reach statistical significance (*p* = 0.079; Figure 5D–F).

### 3.6. Low Dose of Anti-DBH-Saporin Leads to an Increase in Tau Phosphorylated at Thr 217 in the Cortex

**Figure 6:**
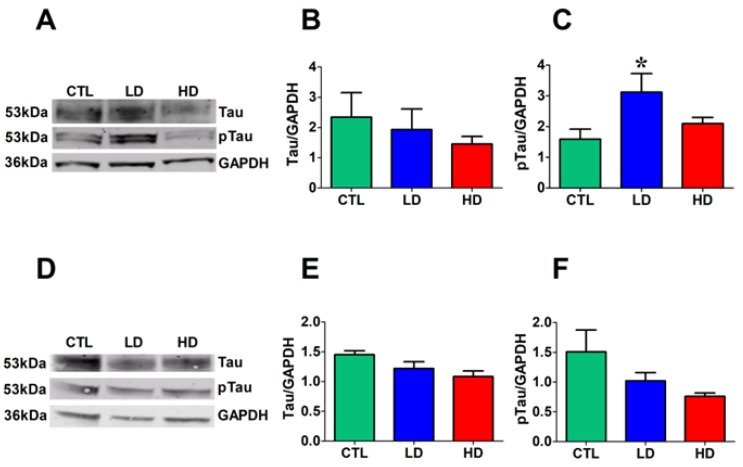
Increased steady-state levels of pTau in the cortex of LD rats. (**A**) Representative Western blot bands of proteins extracted from the cortex of the 3 groups. Densitometric analysis of Tau (**B**) and pTau (**C**) levels of proteins extracted from the cortex. (**D**) Representative Western blot bands of proteins extracted from the hippocampus of the 3 groups. Densitometric analysis of Tau (**E**) and pTau (**F**) levels of proteins extracted from the hippocampus. Data were obtained by normalizing our protein of interest to GAPDH loading control. Asterisk indicates significant differences from CTL group (*p* < 0.05).

Increases in CSF and plasma levels of Tau phosphorylated at Thr 217 have been reported in MCI, which evolves into AD [7,8,9]. To validate the LD group as a possible model of MCI, cortical and hippocampal levels of Tau phosphorylated at Thr 217 were measured by Western blot analysis. Again, one-way ANOVA and the Tukey post hoc test revealed no group differences in the cortical (Figure 6A,B) or hippocampal (Figure 6D,E) total Tau levels. By contrast, phosphorylated Tau was significantly increased in the cortex (Figure 6A,C) of the LD group compared to CTL (*p* < 0.05); however, no such changes were detected in the hippocampus (*p* = 0.198) or in any region of the HD group (Figure 6D,F).

## 4. Discussion

AD is a neurodegenerative disease characterized by a progressive loss of cognitive functions and accumulation of pathological proteins [50]. The pathophysiological mechanisms leading to its onset are not certain. Cumulative evidence indicates that neurotransmitter dysfunction, an event that occurs in the initial stages of the disease, might lead to memory loss and accumulation of pathological proteins [51]. Degeneration of LC neurons and their noradrenergic projections to the neocortex and hippocampus occurs already in MCI patients, and this loss intensifies with the progression of the disease [17,19,52,53]. In addition, the progressive loss of LC neurons is associated with an exacerbation of cognitive decline, especially in episodic and working memory [20]. To further examine the impact of selective NA depletion on spatial learning and memory and to address the possible relationship between these alterations and the development of AD-like neuropathology, we generated a model of mild and severe noradrenergic depletion induced early after birth in rats, using increasing doses of the selective noradrenergic immunotoxin anti-DBH-saporin. Consistent with previous observations, noradrenergic depletion caused working memory deficits in a dose-dependent manner, whereas reference memory abilities were unaffected. Considering the role of NA as a major modulator of cognitive functions such as attention, motivation and memory, these data suggest that some of the learning and memory deficits observed in the early stages of AD, particularly those related to working memory, might be causally associated with the loss of LC neurons [17,23]. These results are not meant to imply that the loss of LC neurons is the only cause of cognitive decline in AD patients, but rather that it may represent one of the earliest events that contribute to cognitive decline before any other pathological feature appears. This early loss of LC neurons also suggests that this neuronal population is more vulnerable than other neuronal types to neuropathological changes in the brain of AD patients [54]

TDP-43 is a nuclear protein encoded by the TARDBP gene on chromosome 1 [55,56]. Pathologic TDP-43 is mislocalized from its nuclear location to the cytoplasm, where it accumulates and forms toxic inclusions that are found in frontotemporal dementia (FTD), amyotrophic lateral sclerosis (ALS) and 36–56% of AD cases [1,11,12]. TDP-43 can also be subjected to post-translational modifications, and in particular, phosphorylated TDP-43 (pTDP-43) is considered a marker of the disease and used as a diagnostic tool to identify TDP-43-positive protein inclusions in the brain and spinal cord of ALS and FTD patients [57,58,59,60]. Moreover, pTDP-43 is more prone to aggregation [15], and TDP-43 phosphorylated at Ser 409/410 and 403/404 accumulates in AD brains [16]. In a previous study, we found that the NA-depleted hippocampal tissue increased the amount of cytosolic TDP-43, but this event was reversed after noradrenergic reinnervation of the hippocampus [37]. However, it is still unclear whether the loss of noradrenergic neurons and fibers contributes to an increase in pTDP-43 levels. Interestingly, we found a significant increase in pTDP-43 levels in the cortex of the HD group. Furthermore, we found a strong trend for an increase in pTDP-43 levels in the hippocampus of the HD group. In the LD group, we did not find any increase in the levels of pTDP43 even though the rats had deficits in working memory. This inconsistency may be caused by the timing and progression of the noradrenergic fiber loss. In cases where the loss is relatively mild or occurs slowly over time, the brain may have sufficient time to activate compensatory mechanisms, altogether leading to an upregulation of protein clearance pathways, known to be under NA control [22], to prevent an increase in the levels of toxic proteins. Additionally, the specific neurodegenerative processes associated with toxic protein accumulation may not be triggered by noradrenergic fiber loss alone, but, rather, they might require additional factors to manifest [56,61,62]. Overall, further studies are needed to fully understand the intricate interactions between the noradrenergic system and the development of pTDP-43 in AD. These data suggest that the most severe depletion of the noradrenergic fibers in the hippocampus and cortex leads to an increase in the steady-state levels of the pathological pTDP-43. To our knowledge, this represents the first evidence of a possible association between lesion-induced noradrenergic dysfunction and increased levels of pTDP-43 in the brains of experimental animals. This finding might be highly relevant to AD neuropathology, given the prominent role of pTDP-43 in AD [11], and warrants further investigation.

Tau is a soluble protein encoded by the MAPT gene localized on chromosome 17 whose primary function is to stabilize microtubules [63,64]. In AD, Tau is hyperphosphorylated at selective amino acids; phosphorylated Tau has a reduced affinity for microtubules, which leads Tau to aggregate and form insoluble NFTs [65]. The accumulation of Tau in the LC is an early event of AD pathogenesis, and as the disease progresses, Tau spreads and accumulates throughout the brain [27,28]. Phosphorylated Tau is often used as a biomarker of AD. For example, pTau T217 and pTau T181 have been detected in the CSF and blood of patients with AD [9]. pTau T217 is considered a highly specific biomarker for the detection of both preclinical (MCI) and advanced AD [7]. Here we report that a relatively mild noradrenergic depletion increases the levels of pTau T217, suggesting that it may contribute to the accumulation of pathological Tau in the cortex, but the same seemingly does not apply following a near-complete noradrenergic loss. The reason for such discrepancy is unclear: a possible explanation could be that a mild loss of noradrenaline may trigger specific compensatory responses that in turn activate cellular pathways leading to the modulation of tau protein expression. However, when the loss of noradrenaline becomes more severe, the cellular responses or compensatory mechanisms may be overwhelmed or saturated, resulting in no further significant changes in tau levels [64,65]. Alternatively, but not necessarily in contrast, it is also possible that noradrenaline levels have a dose-dependent effect on tau protein regulation. A mild loss of noradrenaline might trigger specific signaling pathways or molecular events that modulate tau levels, while a stronger loss would engage different or additional mechanisms that counteract the changes in tau protein expression. The complexity of these dose-dependent effects could contribute to the observed differences and needs to be investigated further.

Notably, the levels of pTau T217 were unaffected in the hippocampus for both the LD and HD groups. The reason for such findings could be attributed to factors such as the non-progressive nature of the noradrenergic lesion adopted here, the time points of analysis, or other factors. It is important to note that the development and progression of neurodegenerative diseases like AD involve a complex interplay of multiple factors, including genetic predisposition, protein misfolding, inflammation and synaptic dysfunction, among others. Although noradrenergic dysfunction may contribute to the overall neurodegenerative process, additional factors and pathways are likely involved in the accumulation of pTau in the hippocampus.

The phosphorylation of tau and TDP-43, two proteins associated with different neurodegenerative conditions, becoming altered due to changes in the noradrenergic system highlights the intricate connections between various biological pathways and their potential impact on brain health. While Tau and TDP-43 are distinct proteins with different cellular roles, mounting evidence suggests that their interactions and dysregulation play a crucial role in the pathogenesis of AD [14,66]. In fact, elevated phosphorylated TDP-43 in AD patients has been associated with more severe AD pathology. A study by Josephs et al. found that the link between pTDP-43 in the hippocampus and accelerated hippocampal atrophy was restricted to cases with higher Braak stages (III-VI) [67]. This indicates that there might be a mutual influence between tau and TDP-43, contributing to neuronal dysfunction and neurodegeneration [68]. Research suggests that TDP-43 might interact with Tau in disease states. Two independent studies performed on transgenic mice and on C. elegans showed that overexpressing TDP-43 specifically enhanced tau but not Aβ and that this resulted in neuronal dysfunction, accumulation of pathological tau and neurodegeneration [14,69]. One of the mechanisms proposed is that TDP-43 could sequester RNA-binding proteins, including those involved in Tau mRNA splicing, leading to altered Tau expression and potentially contributing to Tau pathology [70].

Recently, it was also discovered that both proteins are phosphorylated by TTBK1, a kinase upregulated in AD and in FTLD, further connecting these proteins’ dysregulation. Taylor and colleagues showed that coexpression in C. elegans of tau/TTBK1 or TDP-43/TTBK1 transgenes simultaneously caused a notable amplification of behavioral abnormalities and heightened phosphorylation of pathological proteins ultimately causing cell loss [71]. Dysfunctional phosphorylation events in turn could influence the mislocalization, aggregation and toxicity of these proteins [15,72].

Understanding the complex interactions between Tau and TDP-43 is crucial for unraveling the underlying mechanisms of neurodegenerative diseases. These interactions may represent points of convergence in disease pathways, where aberrations in one protein’s behavior influence the behavior of the other. Targeting these interactions could hold promise for developing therapeutic strategies aimed at mitigating the progression of diseases like Alzheimer’s, FTD, and ALS, where Tau and TDP-43 play central roles in pathology.

## 5. Conclusions

The understanding of the relationship between noradrenergic dysfunction, LC lesions and pTau and pTDP-43 accumulation in the hippocampus is still evolving. Further research is needed to elucidate the underlying mechanisms and to establish a clear causal relationship between these factors. Regardless, it is of interest that the present findings are, at least in part, consistent with studies showing an increase in CSF and plasma levels of pTau T217 in MCI patients that eventually evolved into AD [7,8,9]. The LD group exhibited slightly less severe cognitive impairments and no changes in pTDP-43 levels compared to the HD group, while the increase in pTau T217 was already evident in the LD group. Therefore, the HD group in the present study might be considered a model of more advanced AD, while the LD group may represent a model of MCI. Overall, our finding clearly suggests that preserving normal levels of NA in the central nervous system could be pivotal for preventing or delaying the development of AD.

## Figures and Tables

**Table 1:** Motor tests were administered to all animals at about 6 weeks post-lesion. Numbers represent the mean of four determinations ± SEM.

*Groups*	*Equilibrium Time on Ramp (%)*	*Latency to Cross Ramp (sec)*	*Latency to Reverse on Grids (sec)*	*Number of Falls in Grids*
CTL (n = 8)	98.6 ± 7.3	6.8 ± 0.4	5.6 ± 0.9	1.8 ± 0.7
LD (n = 8)	98.1 ± 6.6	6.7 ± 0.4	6.1 ± 0.9	3.0 ± 0.5
HD (n = 8)	96.9 ± 13.5	7.2 ± 0.4	6.3 ±0.7	3.5 ± 0.5

**Table 2:** Stereological and densitometric estimates of dopamine-ß-hydroxylase-immunoreactive neurons and fiber density in the locus coeruleus and cortical–hippocampal terminal regions, respectively. Values indicate the estimated number of DBH-positive profiles in the LC/SubC and the relative density scores (±SEM) of DBH-positive innervation in the fronto-partietal cortex (Cx) and in the various subfield of the hippocampus, i.e., Cornu Ammonis (CA) 1 and 3 and the dentate gyrus (DG). Asterisks indicate significant difference from the CTL group (*p* < 0.01).

*Group*	*DBH-ir Neurons in LC/SubC*	*DBH-ir* *Fibers in Cx*	*DBH-ir* *Fibers in CA1*	*DBH-ir* *Fibers in CA3*	*DBH-ir* *Fibers in DG*
CTL (n = 8)	1830.4 ± 70.4	86.8 ± 5.0	64.5 ± 3.9	62.8 ± 4.0	63.4 ± 3.9
LD (n = 8)	529.3 ± 34.6 *	23.6 ± 1.0 *	21.6 ± 1.2 *	22.9 ± 0.9 *	19.8 ± 1.4 *
HD (n = 8)	181.1 ± 24.5 *	8.8 ± 1.0 *	6.7 ± 0.8 *	7.1 ± 1.3 *	8.6 ± 1.1 *

## Data Availability

Not applicable.

## Supplementary Materials

The original and uncut Western blots can be downloaded at https://www.mdpi.com/article/10.3390/biology12091264/s1.
