# Supplementary material for: Selective Noradrenaline Depletion in the Neocortex and Hippocampus Induces Working Memory Deficits and Regional Occurrence of Pathological Proteins
Source: Biology (Basel). 2023 Sep 21;12(9):1264. doi: 10.3390/biology12091264 (PMC10526041; doi:10.3390/biology12091264)
Supplement: Supplementary file 1 [file biology-12-01264-s001.zip › Loading order for western blots Cortex and Hipp.pdf]

The loading order for the western blot of cortex (TDP-43, pTDP43, Tau and PTAU) is the following:

Gel 1:

1. CTL
2. LOW DOSE
3. HIGH DOSE
4. CTL
5. LOW DOSE
6. HIGH DOSE
7. CTL
8. LOW DOSE
9. HIGH DOSE
10. MARKER

Gel 2:

1. Marker
2. CTL
3. LD
4. HD
5. CTL
6. LD
7. HD
8. CTL
9. LD
10. CTL

The loading order for the western blot of Hippocampus (TDP-43, pTDP43, Tau and PTAU) is the following:

1. CTL
2. LOW DOSE
3. HIGH DOSE
4. CTL
5. LOW DOSE
6. HIGH DOSE
7. CTL
8. LOW DOSE
9. LOW DOSE
10. Marker
