# Supplementary figures and images for: Selective Noradrenaline Depletion in the Neocortex and Hippocampus Induces Working Memory Deficits and Regional Occurrence of Pathological Proteins
Source: Biology (Basel). 2023 Sep 21;12(9):1264. doi: 10.3390/biology12091264 (PMC10526041; doi:10.3390/biology12091264)

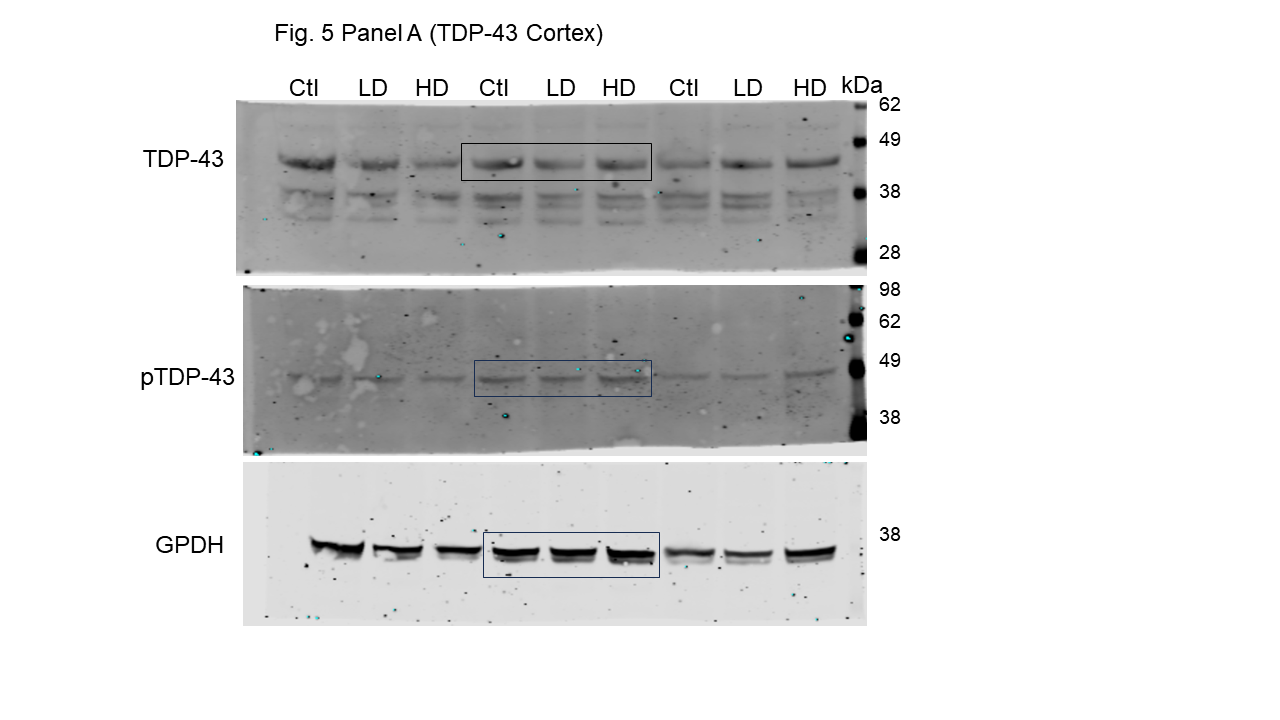

Supplement: Supplementary file 1 [file biology-12-01264-s001.zip › Fig. 5 Panel A.tif]

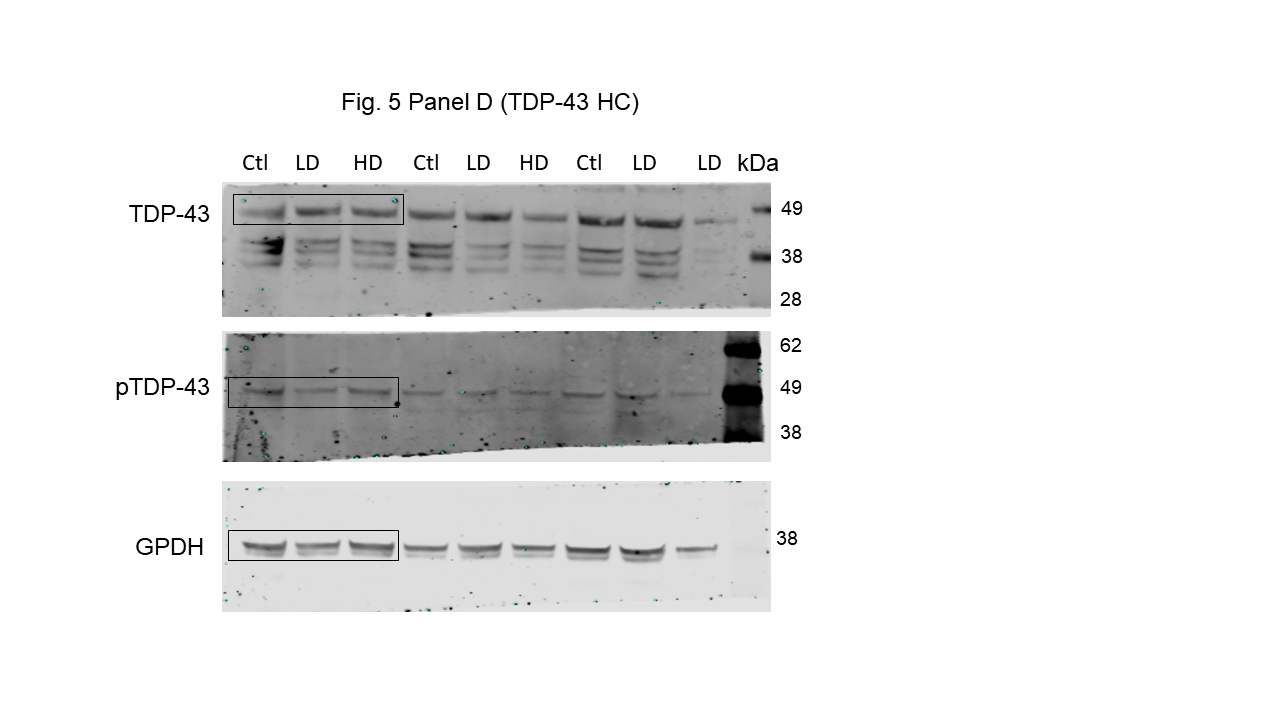

Supplement: Supplementary file 1 [file biology-12-01264-s001.zip › Fig. 5 PAnel D.tif]

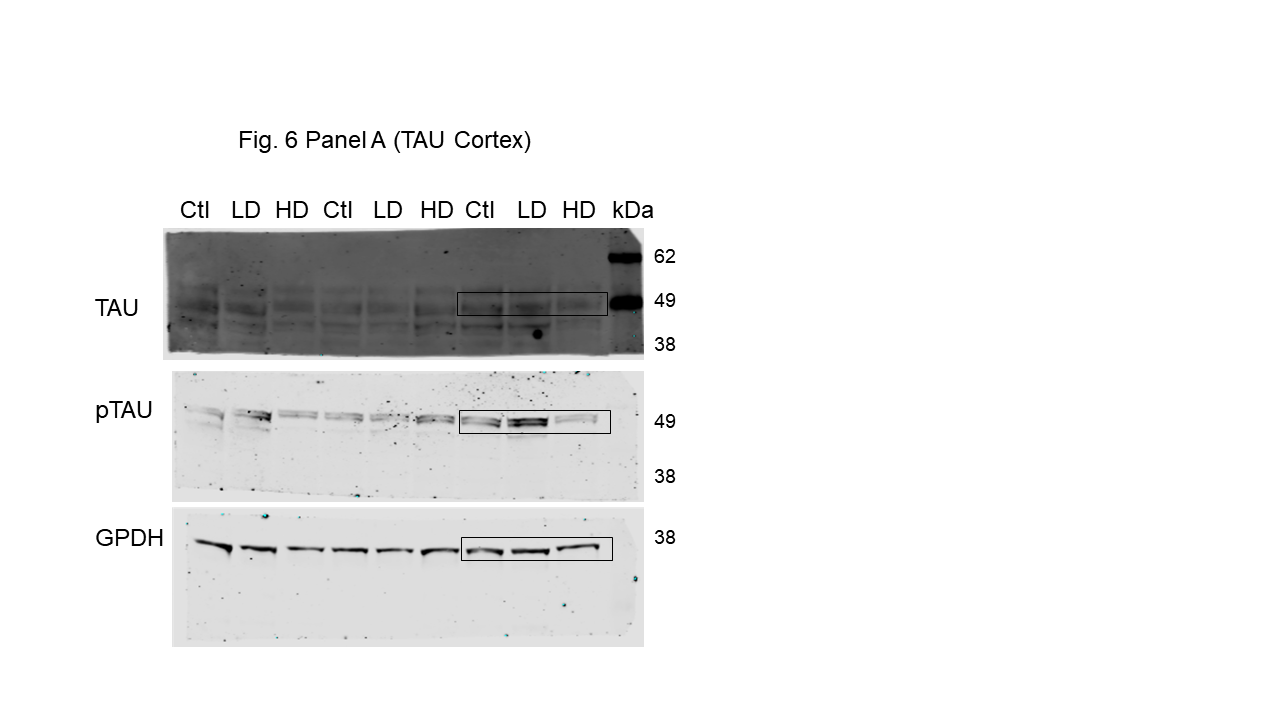

Supplement: Supplementary file 1 [file biology-12-01264-s001.zip › Fig. 6 Panel A.tif]

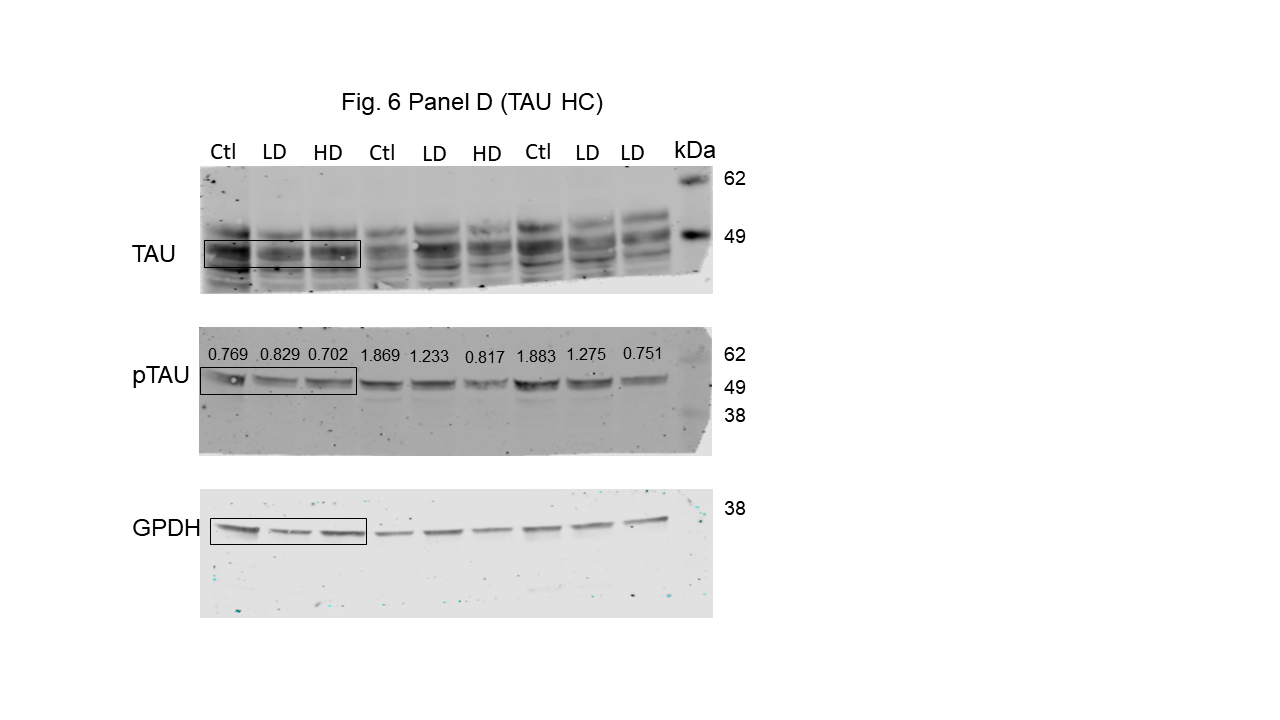

Supplement: Supplementary file 1 [file biology-12-01264-s001.zip › Fig.6 Panel D.tif]

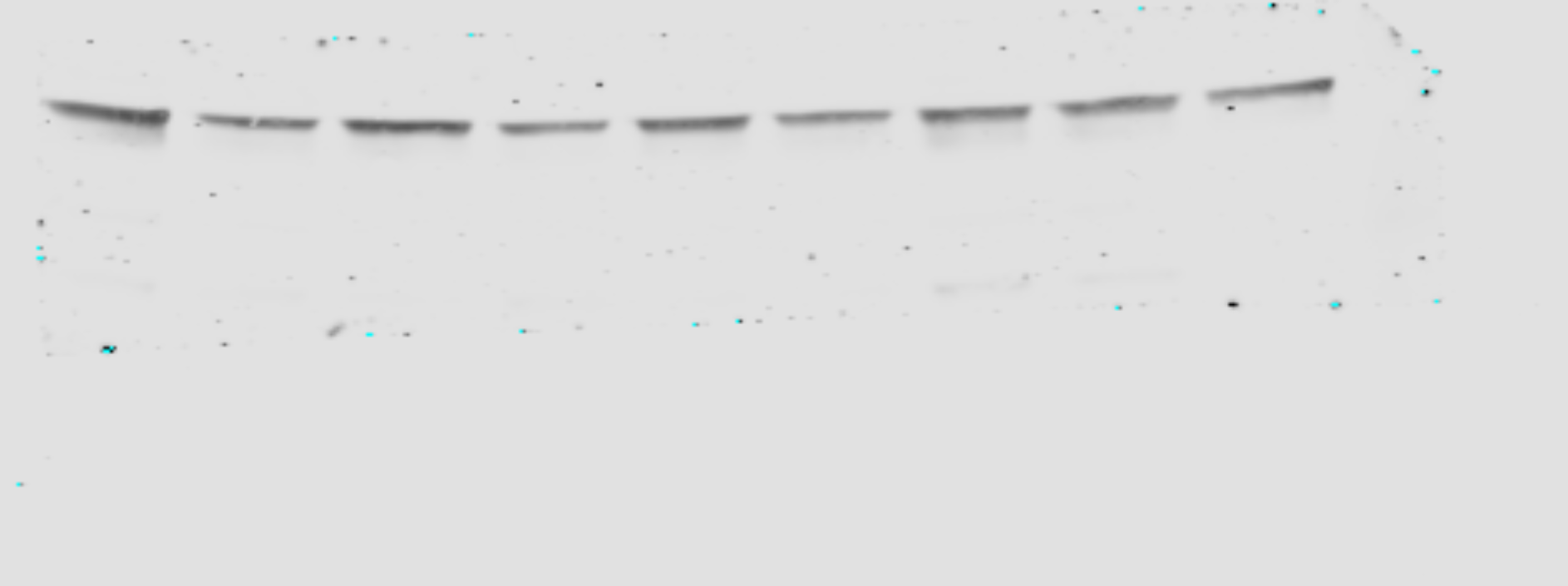

Supplement: Supplementary file 1 [file biology-12-01264-s001.zip › gapdh for TAU HC.tif]

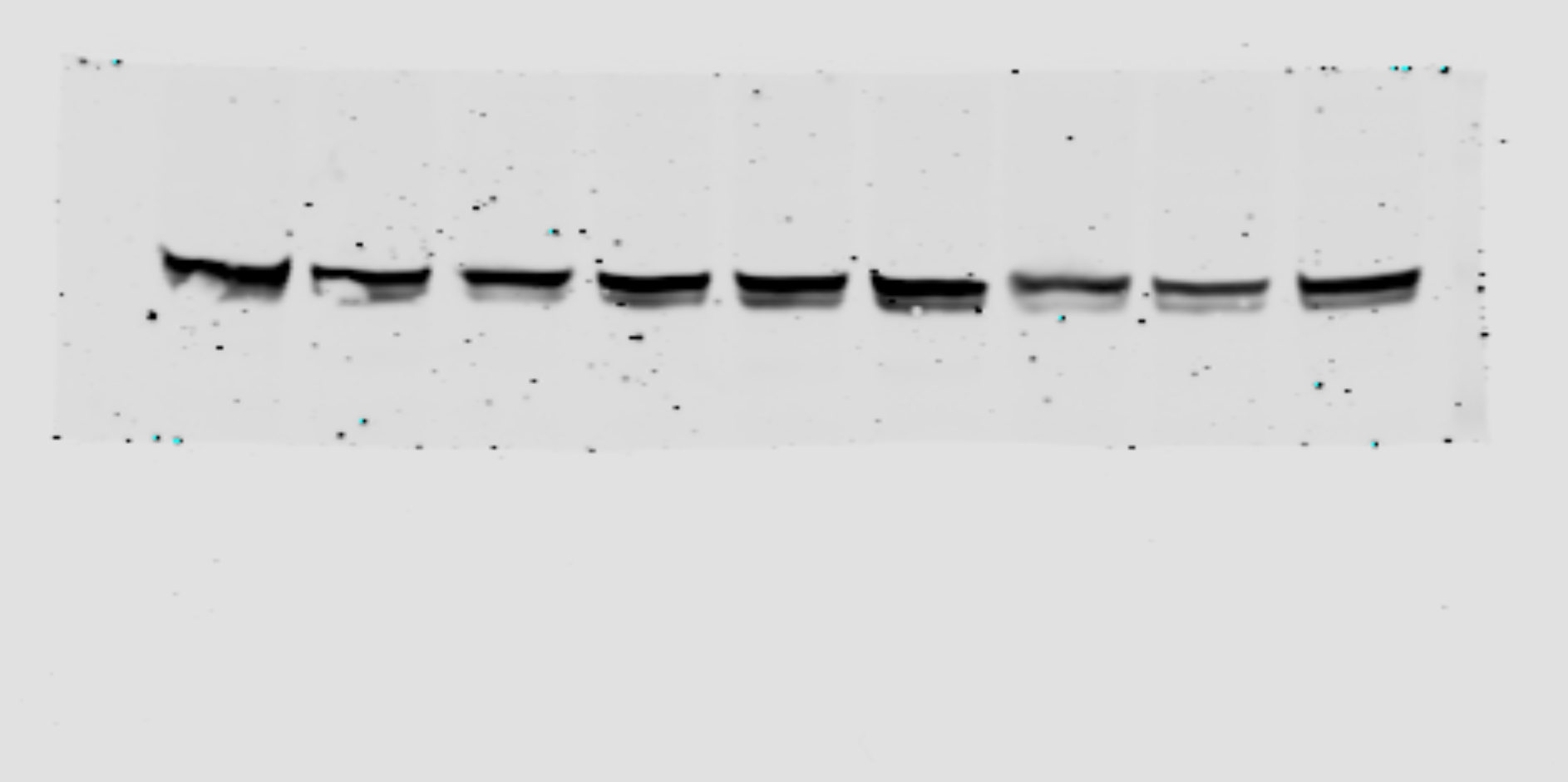

Supplement: Supplementary file 1 [file biology-12-01264-s001.zip › GAPDH for TDP-43 CX gel 1.tif]

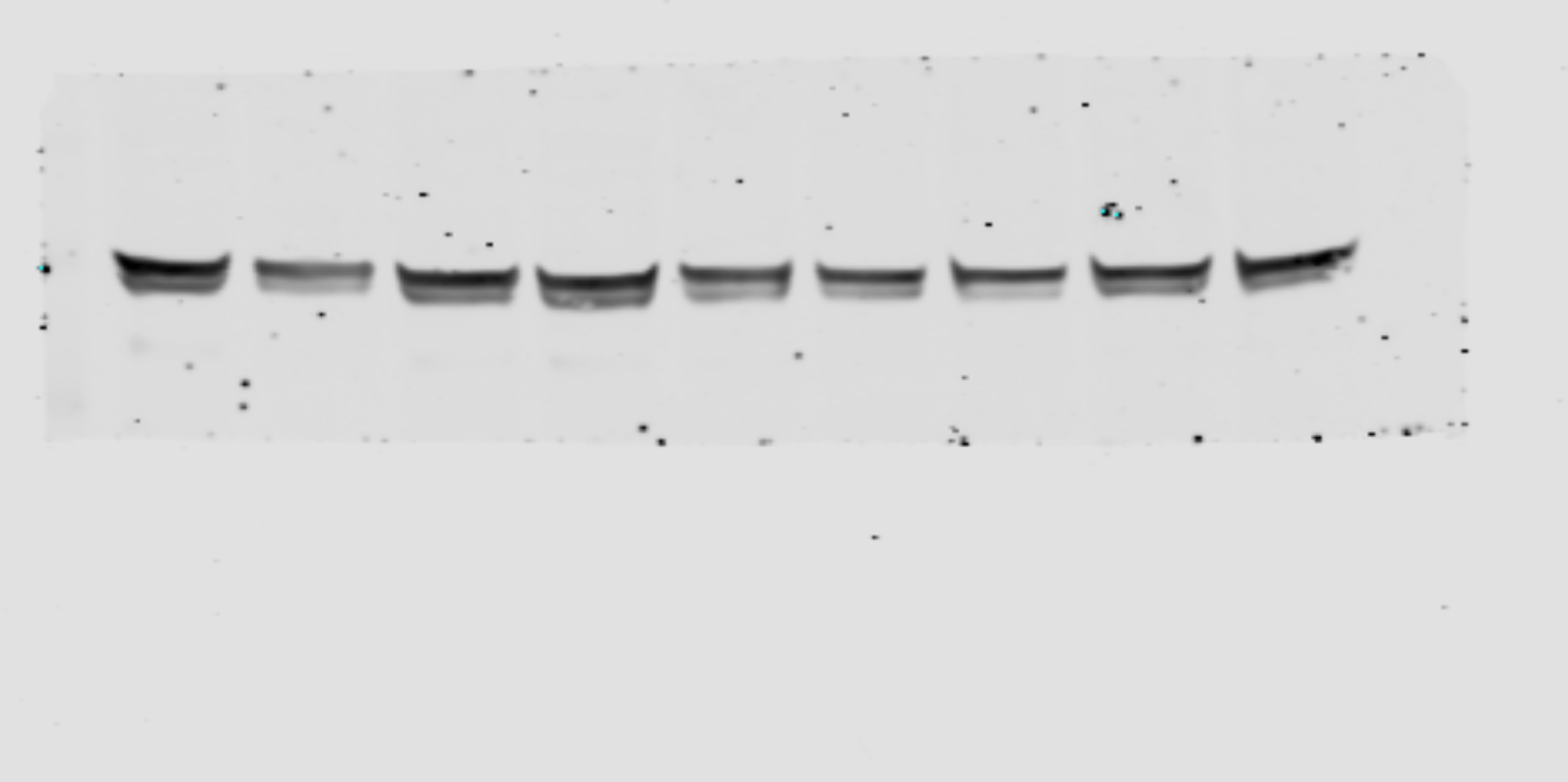

Supplement: Supplementary file 1 [file biology-12-01264-s001.zip › GAPDH for TDP-43 CX gel 2.tif]

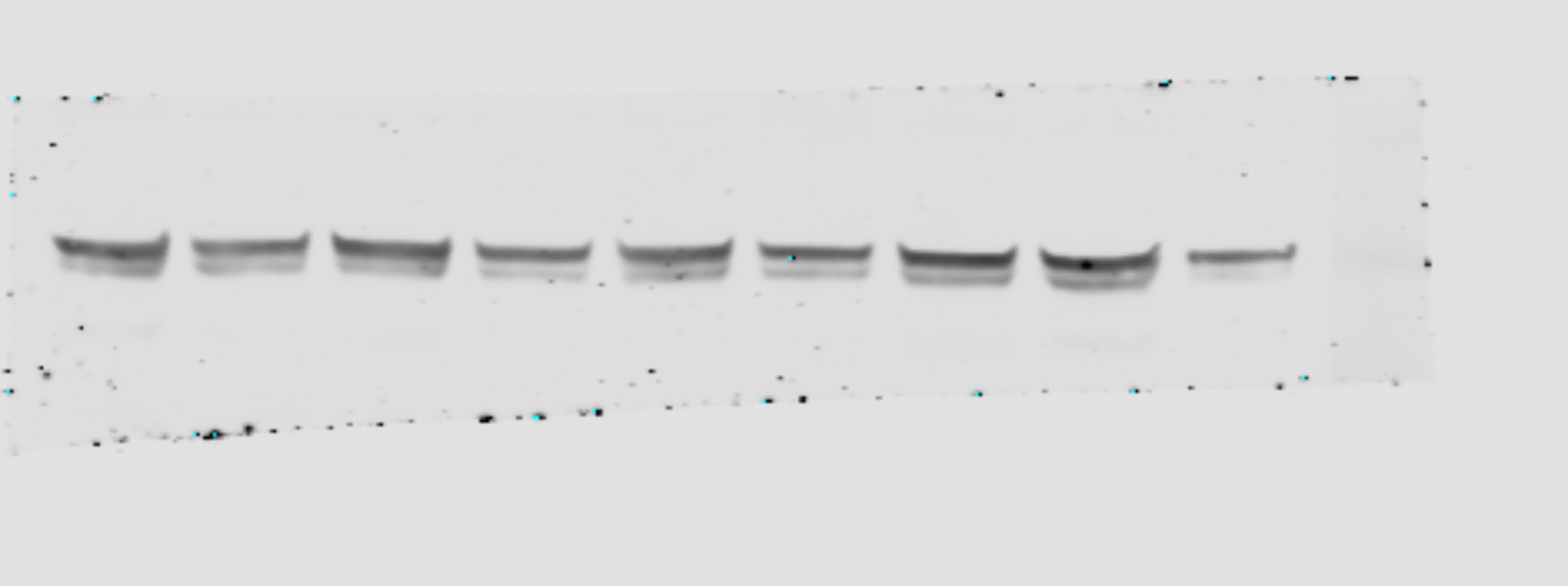

Supplement: Supplementary file 1 [file biology-12-01264-s001.zip › GAPDH FOR TDP43 HC.tif]

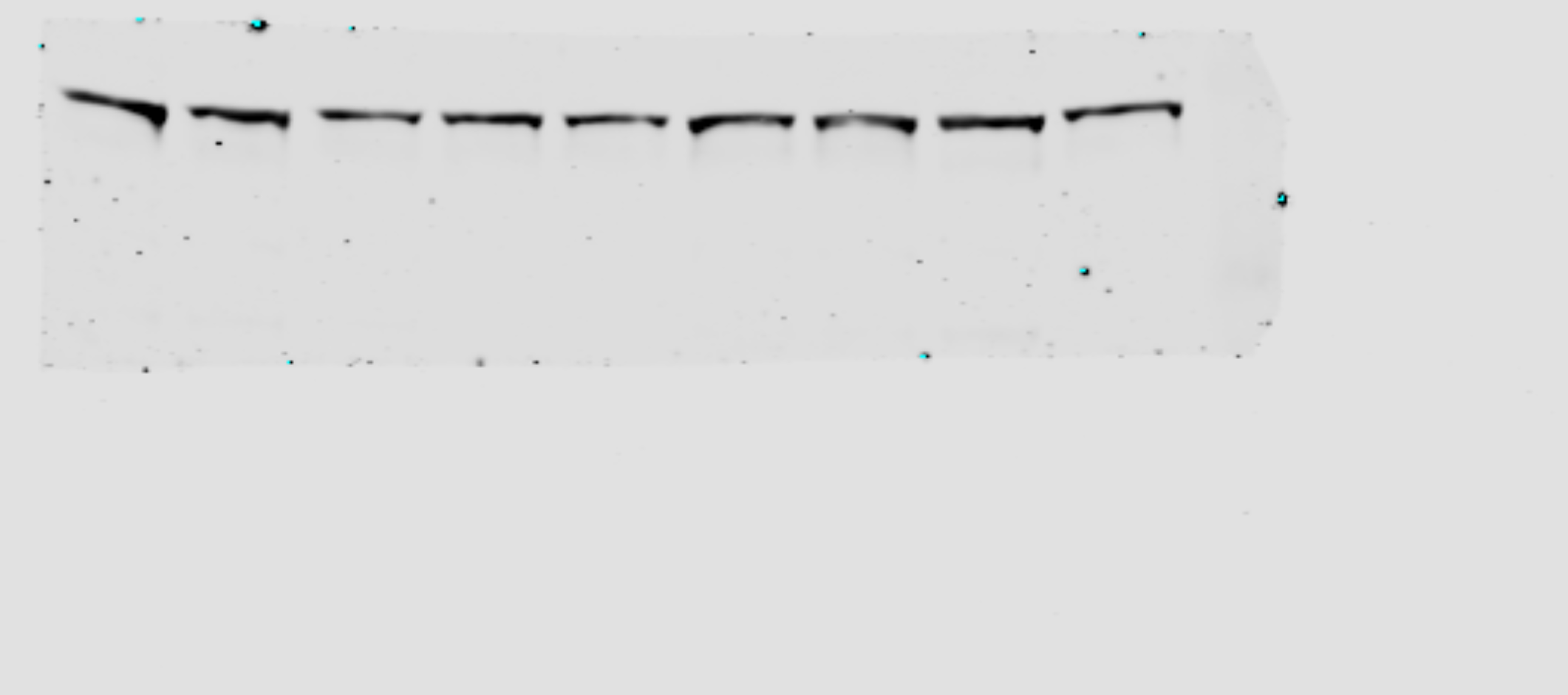

Supplement: Supplementary file 1 [file biology-12-01264-s001.zip › GAPDH x TAU CX gel 1.tif]

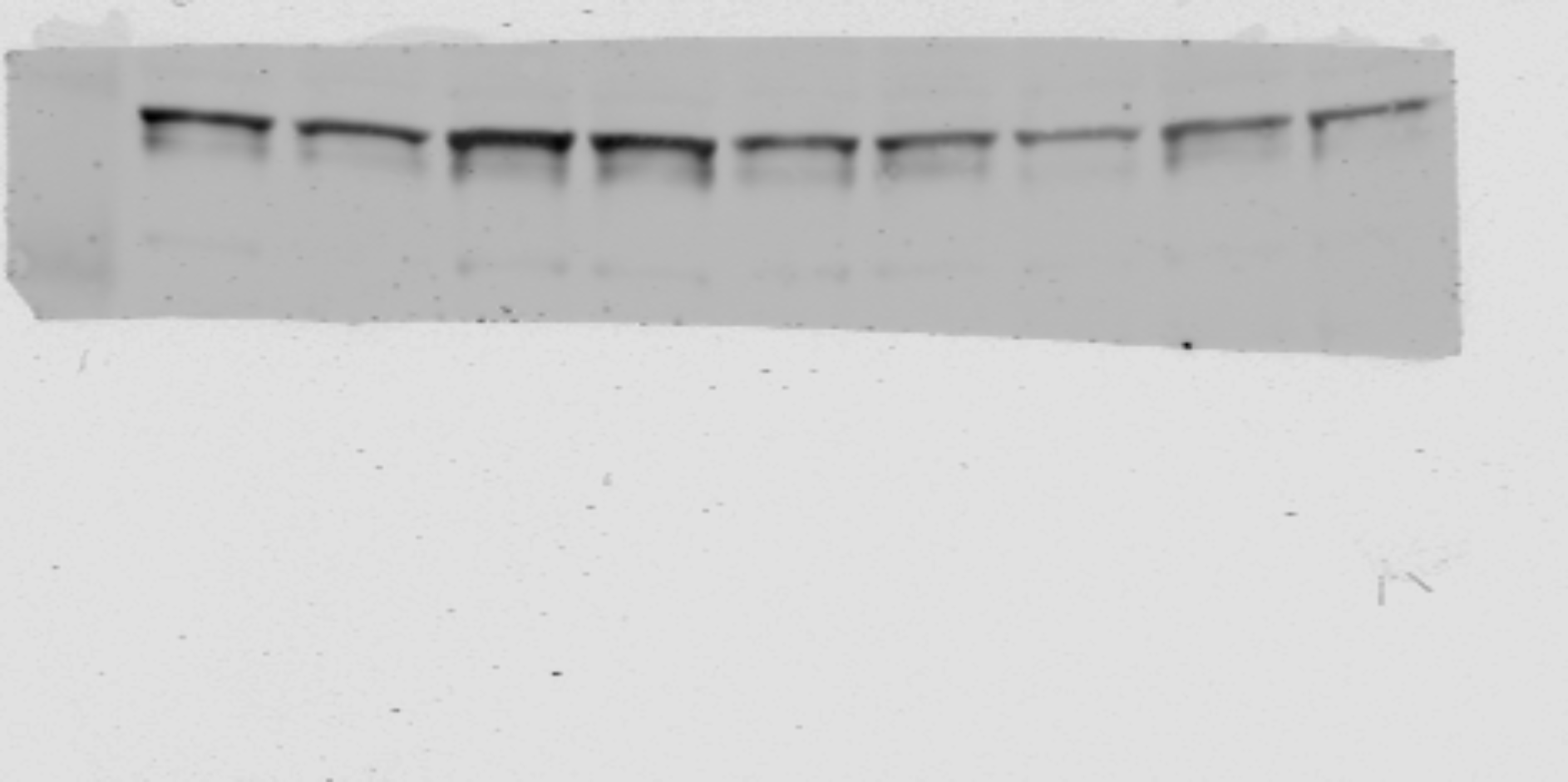

Supplement: Supplementary file 1 [file biology-12-01264-s001.zip › gapdh x TAU CX gel 2.tif]

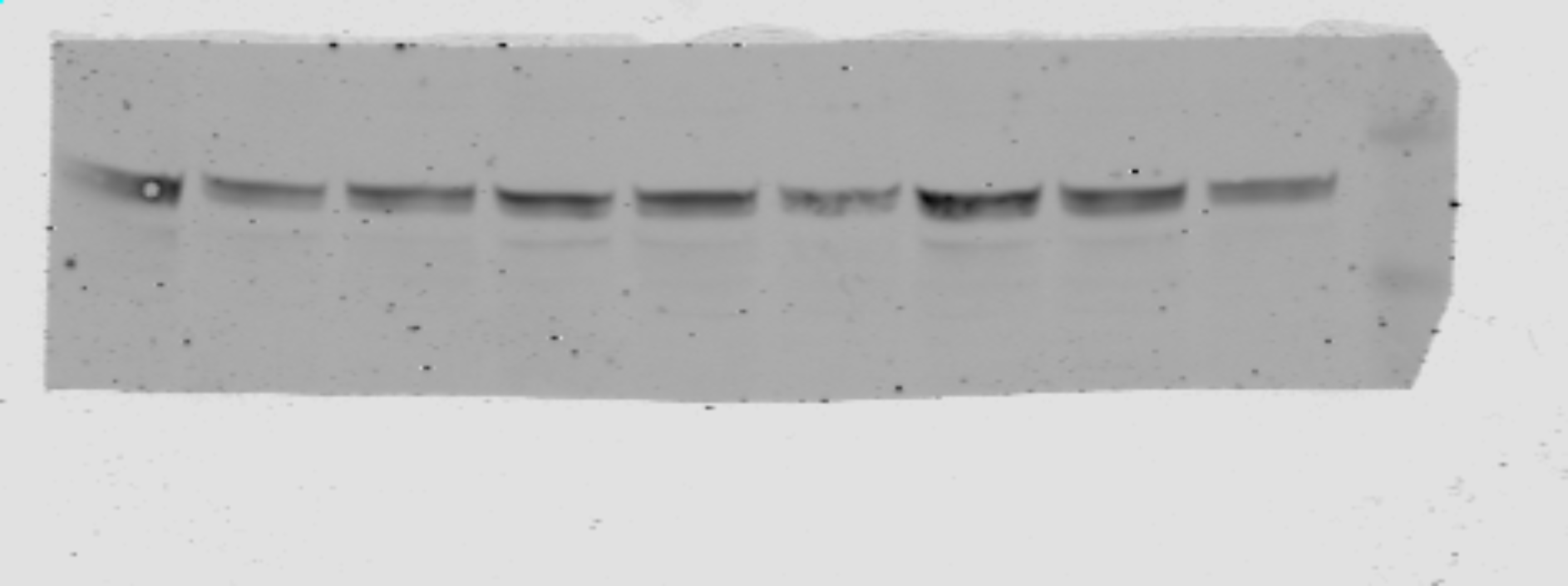

Supplement: Supplementary file 1 [file biology-12-01264-s001.zip › p TAU HC.tif]

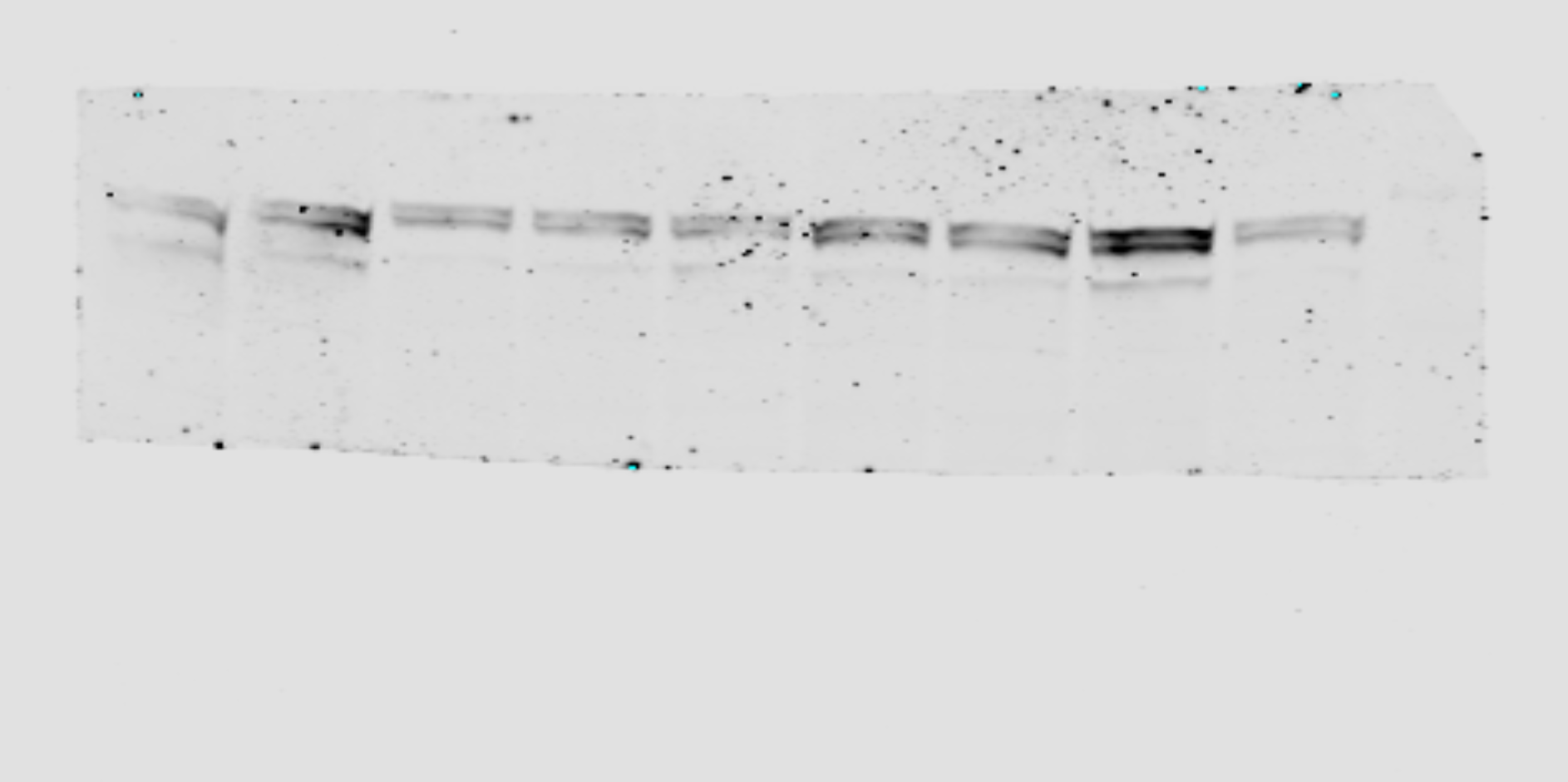

Supplement: Supplementary file 1 [file biology-12-01264-s001.zip › pTAU CX gel 1.tif]

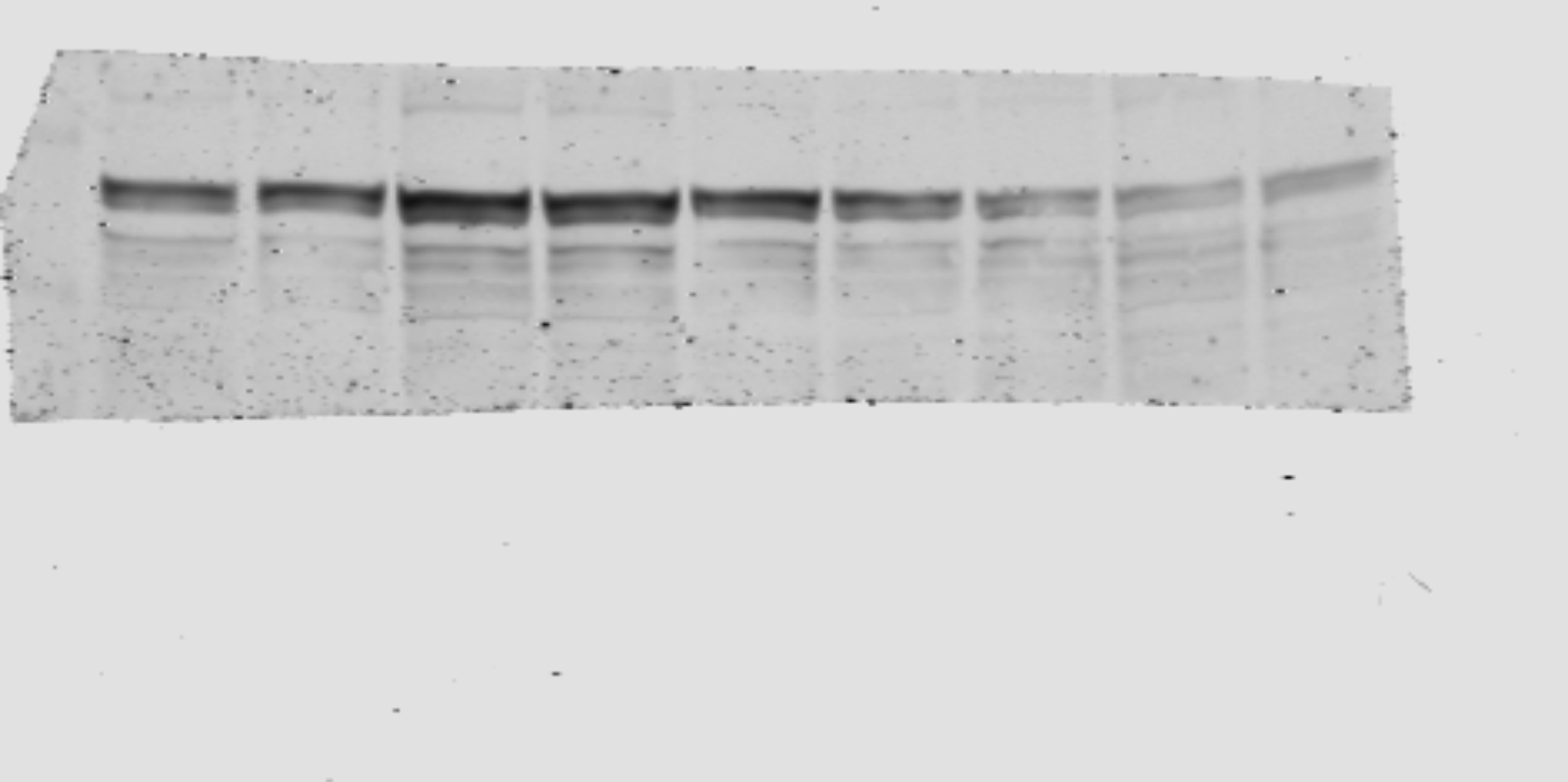

Supplement: Supplementary file 1 [file biology-12-01264-s001.zip › pTAU CX gel 2.tif]

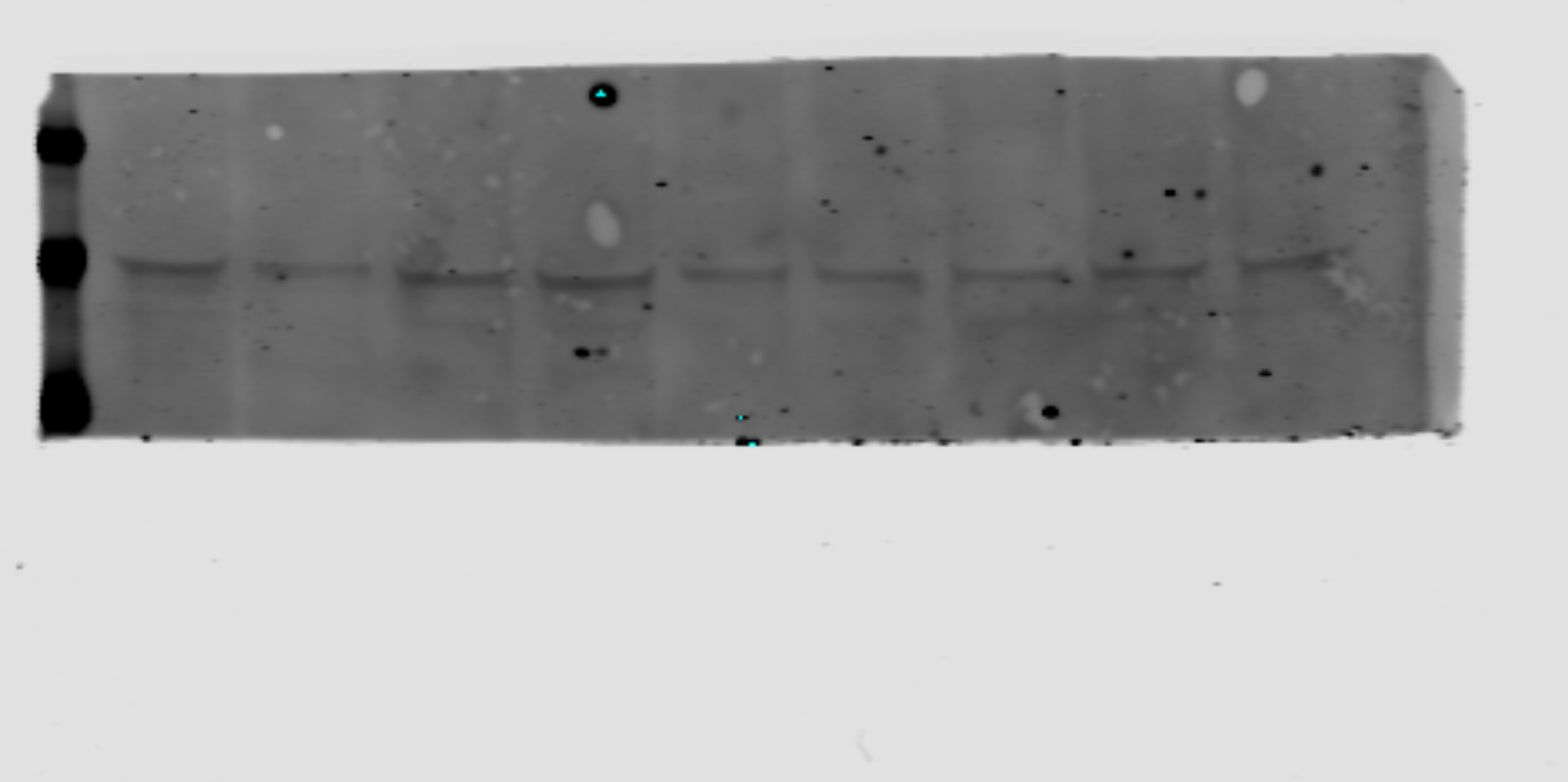

Supplement: Supplementary file 1 [file biology-12-01264-s001.zip › pTDP CX gel 2.tif]

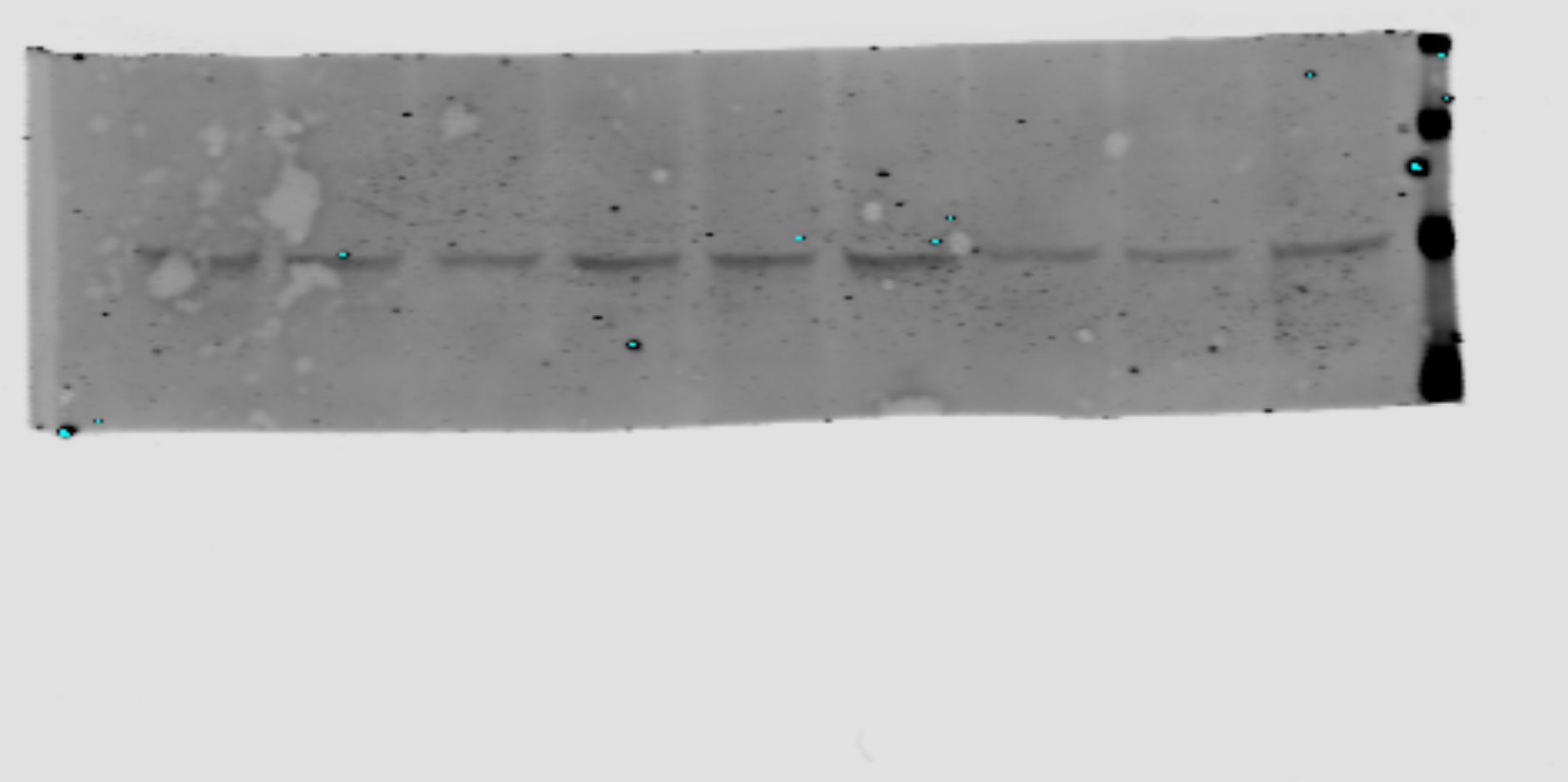

Supplement: Supplementary file 1 [file biology-12-01264-s001.zip › pTDP43 CX gel 1.tif]

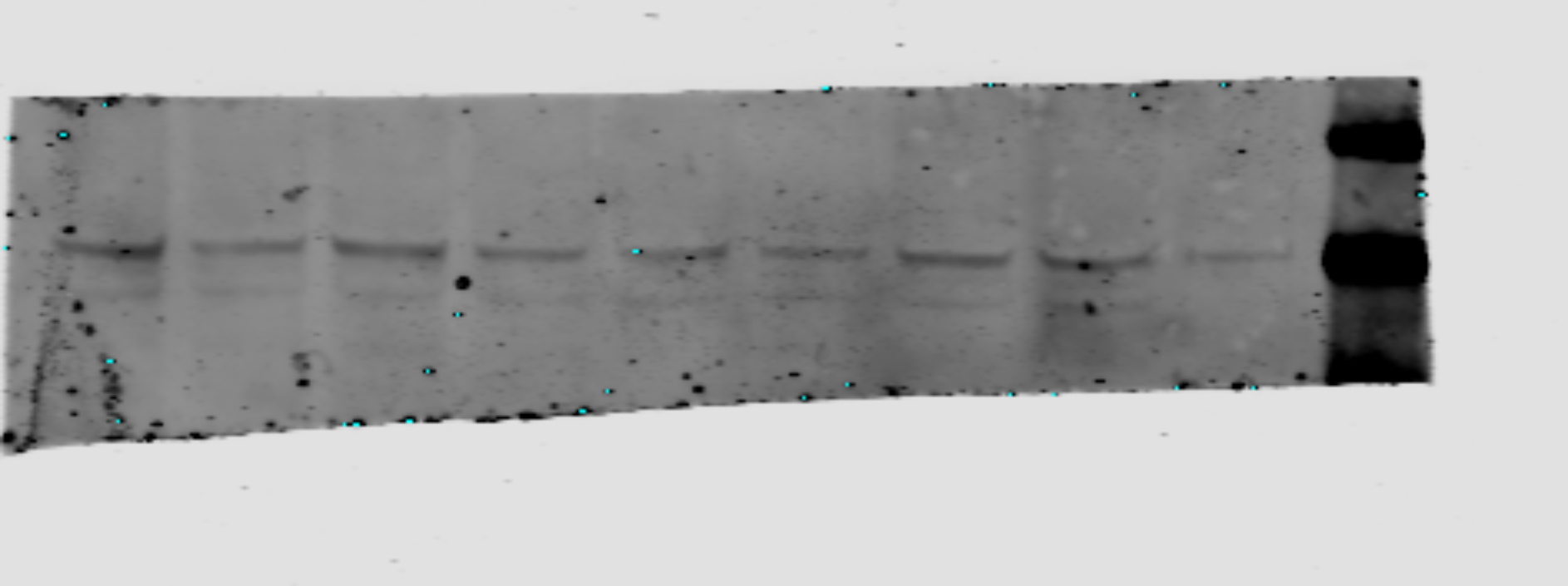

Supplement: Supplementary file 1 [file biology-12-01264-s001.zip › pTDP43 HC.tif]

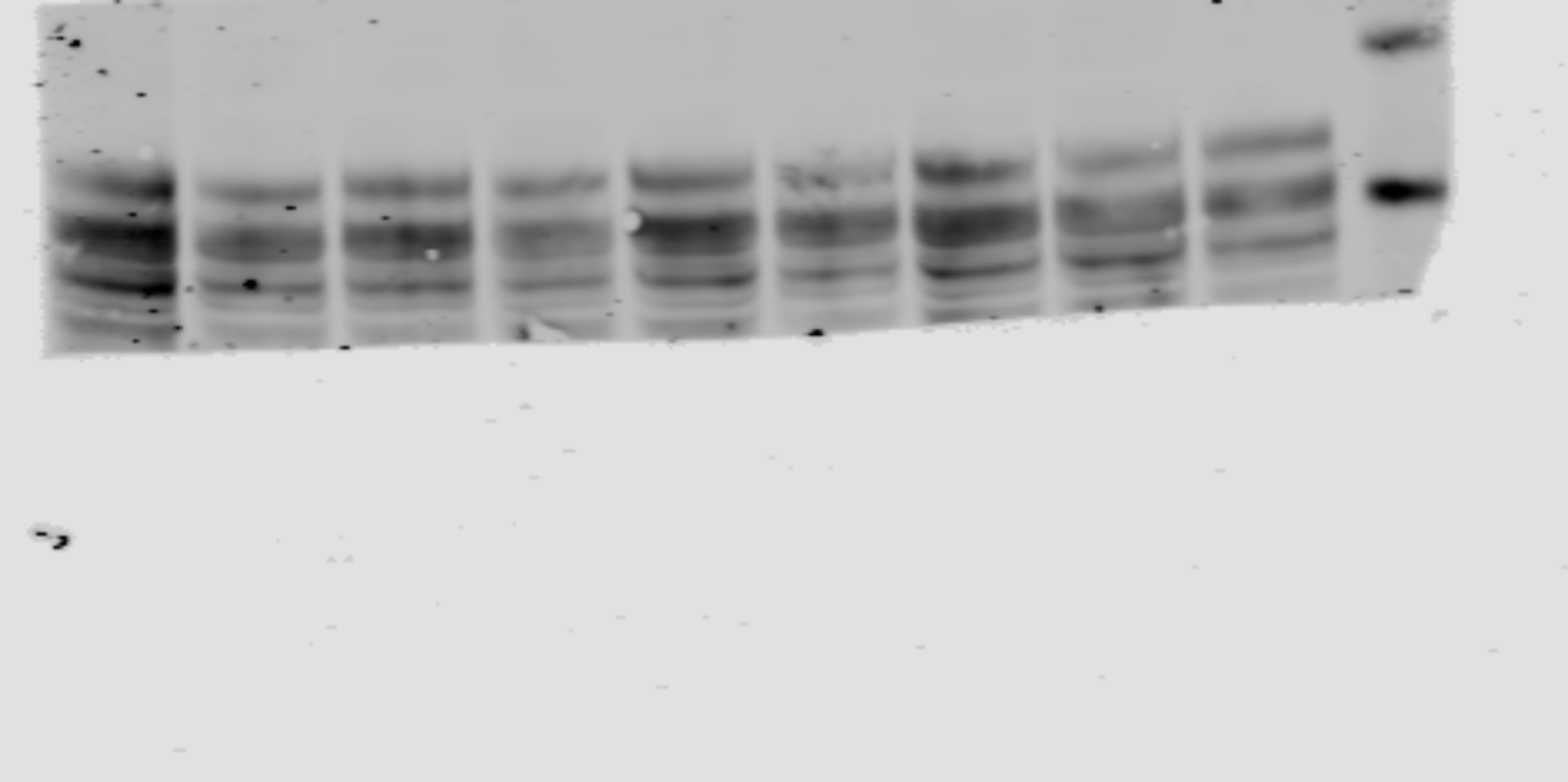

Supplement: Supplementary file 1 [file biology-12-01264-s001.zip › TAU HC.tif]

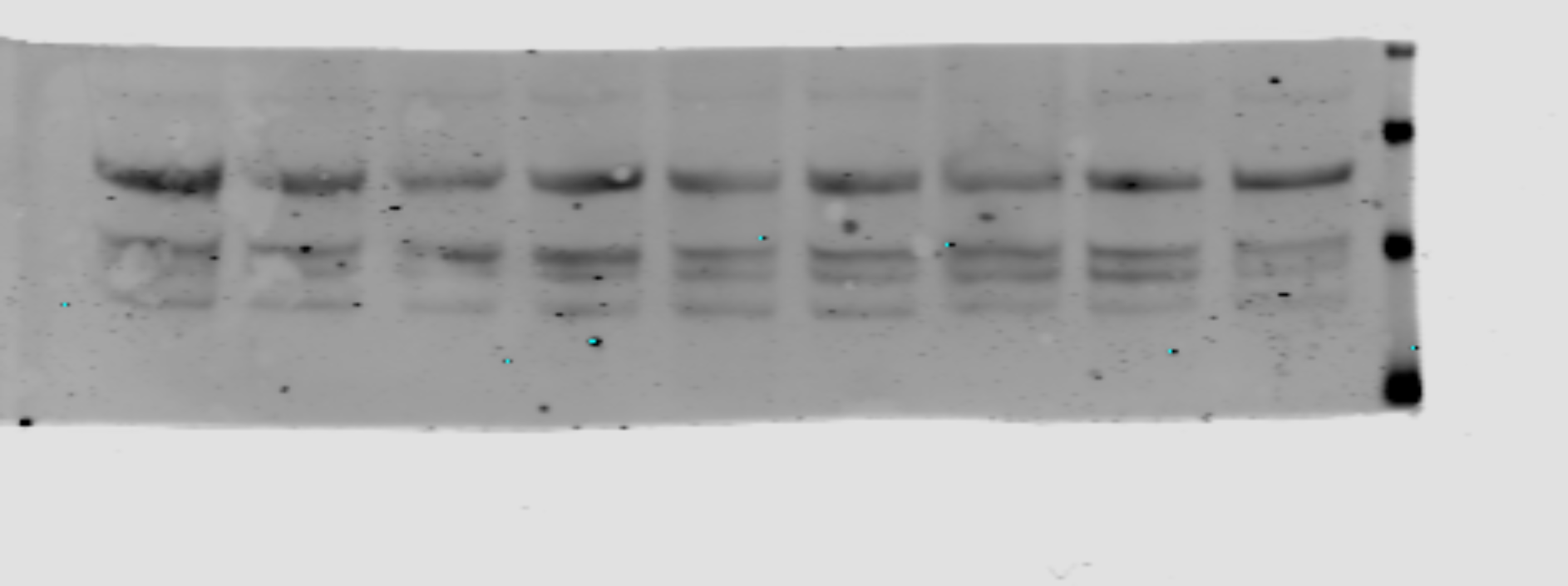

Supplement: Supplementary file 1 [file biology-12-01264-s001.zip › TDP CX gel 1.tif]

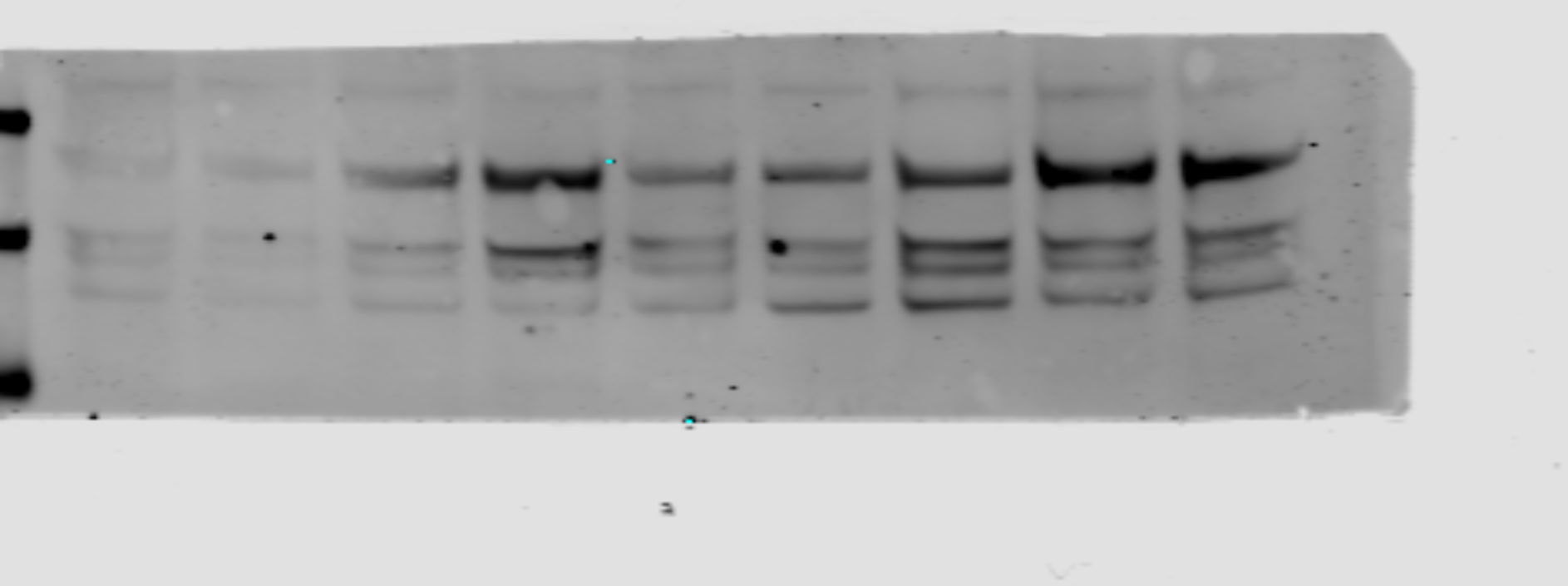

Supplement: Supplementary file 1 [file biology-12-01264-s001.zip › TDP43 CX gel 2.tif]

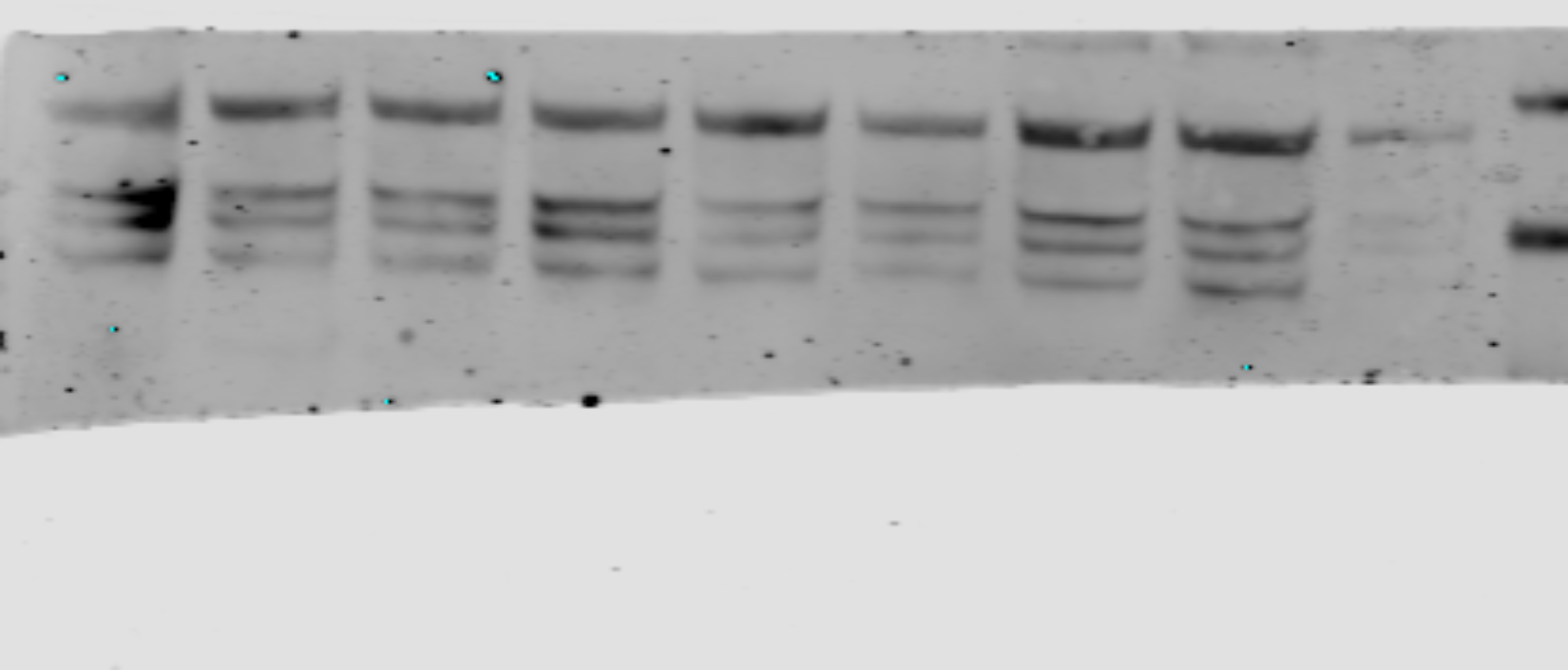

Supplement: Supplementary file 1 [file biology-12-01264-s001.zip › TDP43 HC.tif]

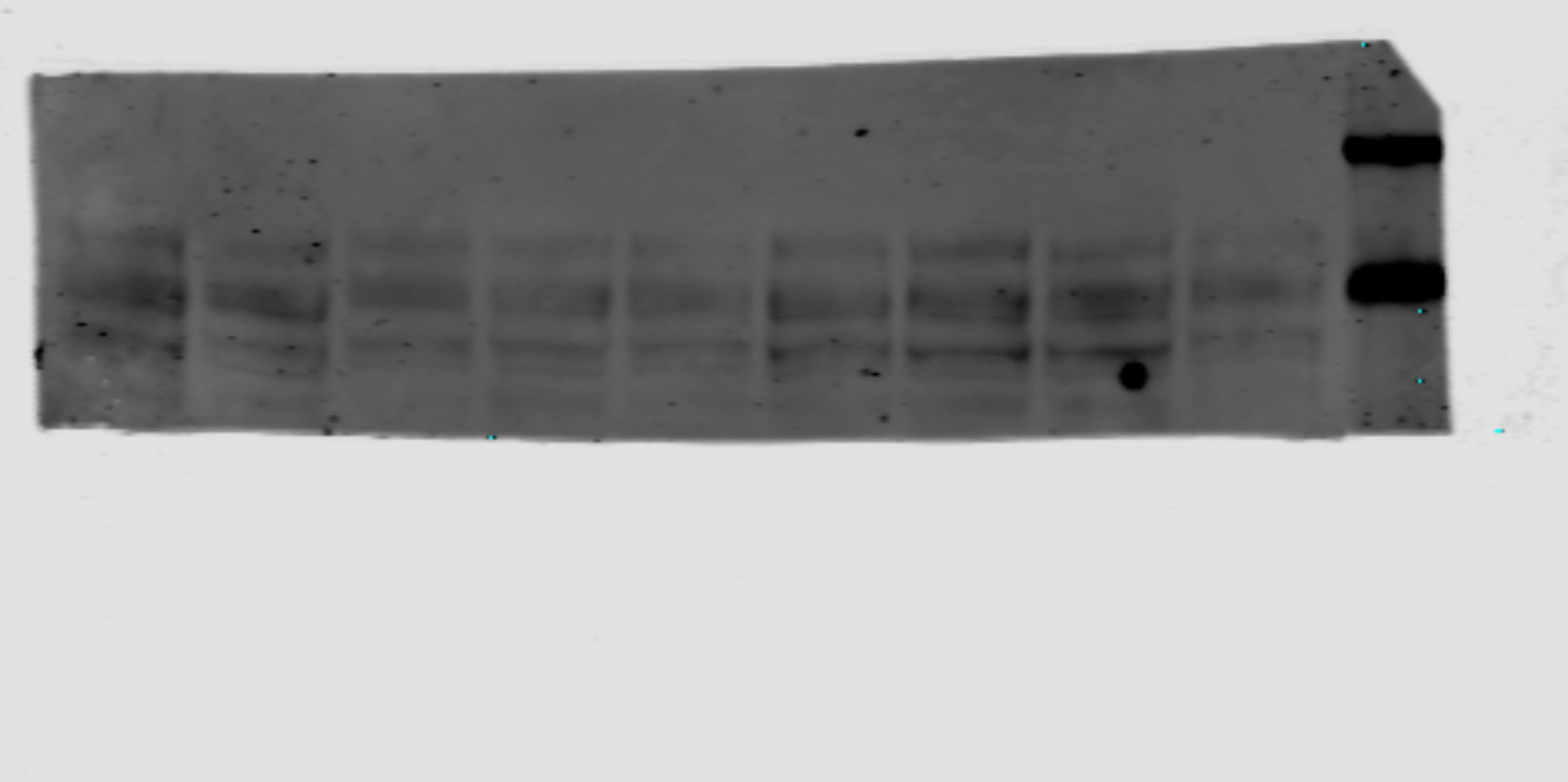

Supplement: Supplementary file 1 [file biology-12-01264-s001.zip › Total Tau CX gel 1.tif]

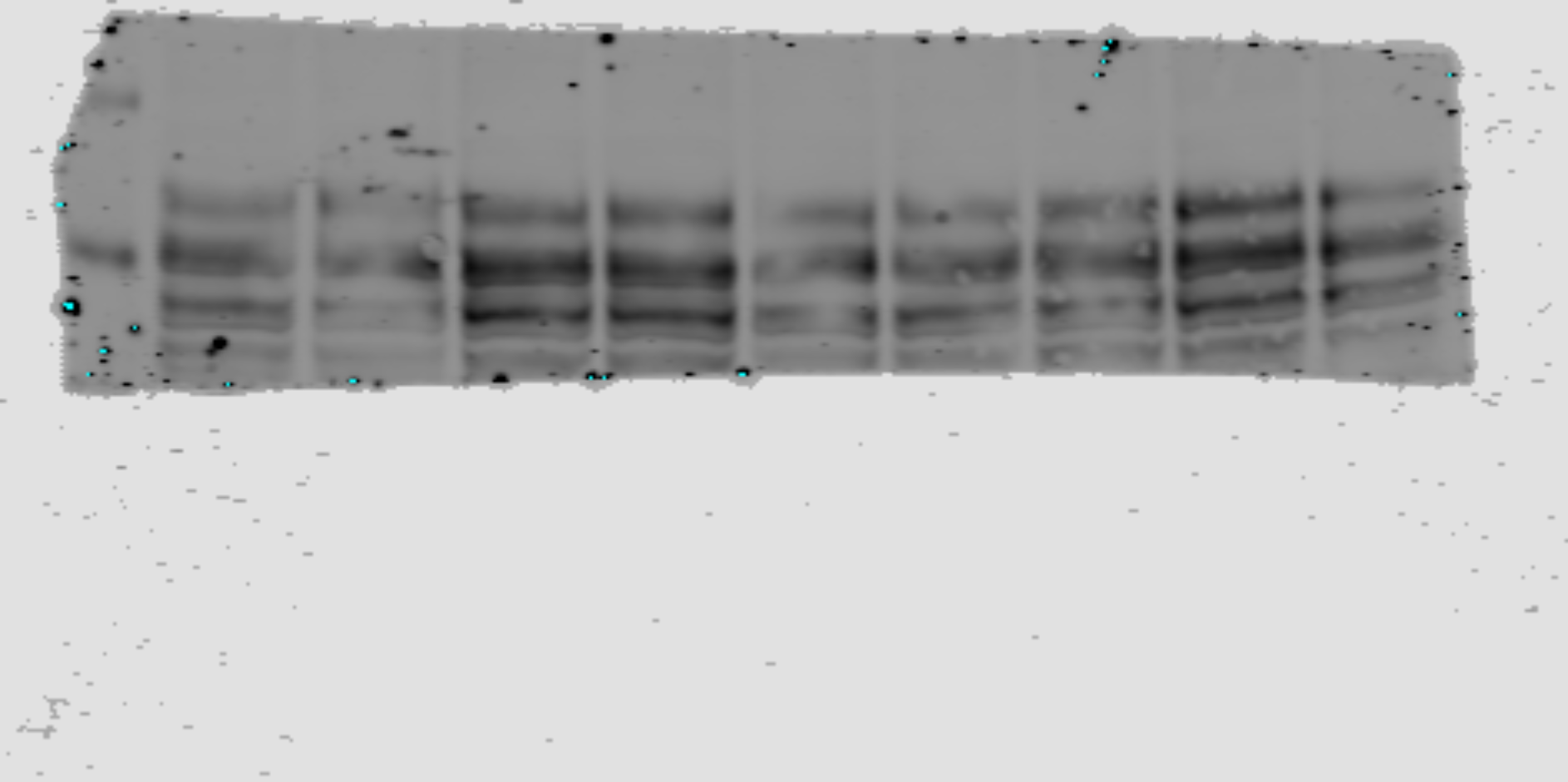

Supplement: Supplementary file 1 [file biology-12-01264-s001.zip › Total TAU CX gel 2.tif]
